# Motion feature extraction using magnocellular-inspired spiking neural networks for drone detection

**DOI:** 10.3389/fncom.2025.1452203

**Published:** 2025-01-22

**Authors:** Jiayi Zheng, Yaping Wan, Xin Yang, Hua Zhong, Minghua Du, Gang Wang

**Affiliations:** ^1^Department of Computer, University of South China, Hengyang, China; ^2^Center of Brain Sciences, Beijing Institute of Basic Medical Sciences, Beijing, China; ^3^Department of Emergency, The First Medical Center, Chinese PLA General Hospital, Beijing, China; ^4^Chinese Institute for Brain Research, Beijing, China

**Keywords:** bio-inspired vision computation, spiking neural networks, motion detection, drone target recognition, motion saliency estimation, visual motion features

## Abstract

Traditional object detection methods usually underperform when locating tiny or small drones against complex backgrounds, since the appearance features of the targets and the backgrounds are highly similar. To address this, inspired by the magnocellular motion processing mechanisms, we proposed to utilize the spatial–temporal characteristics of the flying drones based on spiking neural networks, thereby developing the Magno-Spiking Neural Network (MG-SNN) for drone detection. The MG-SNN can learn to identify potential regions of moving targets through motion saliency estimation and subsequently integrates the information into the popular object detection algorithms to design the retinal-inspired spiking neural network module for drone motion extraction and object detection architecture, which integrates motion and spatial features before object detection to enhance detection accuracy. To design and train the MG-SNN, we propose a new backpropagation method called Dynamic Threshold Multi-frame Spike Time Sequence (DT-MSTS), and establish a dataset for the training and validation of MG-SNN, effectively extracting and updating visual motion features. Experimental results in terms of drone detection performance indicate that the incorporation of MG-SNN significantly improves the accuracy of low-altitude drone detection tasks compared to popular small object detection algorithms, acting as a cheap plug-and-play module in detecting small flying targets against complex backgrounds.

## 1 Introduction

The rapid development of unmanned aerial vehicle technology has led to the wide use of small civilian drones for various tasks such as security patrols, agricultural monitoring, and disaster relief. However, there is also misuse of drones for illegal activities such as smuggling contraband, espionage mapping, and close-range reconnaissance, posing a significant threat to public safety (AL-Dosari et al., 2023). Therefore, it is crucial to develop an early warning detection system for low-altitude, short-range small drones. Traditional radar detection methodologies encounter challenges in identifying small drones due to their limited radar cross-section, low operational altitude, slow velocity, and inclination to conceal within intricate backgrounds, rendering them susceptible to ground clutter interference (Abro et al., 2022). Recent studies have shown the potential of advanced communication and machine learning approaches in improving UAV detection capabilities and reducing interference from complex backgrounds (Khalil et al., 2022). Conversely, optoelectronic sensors, encompassing the infrared and visible light spectra, prove more adept at detecting short-range, low-altitude drone targets in complex settings, and the image and video data it captures need to be further processed using object detection to output results.

Previous research on drone detection has employed various techniques, primarily developed based on deep neural networks. These techniques are classified into two-stage and single-stage algorithms, depending on whether candidate regions are explicitly generated. Two-stage methods, such as the Faster R-CNN (Ren et al., 2015), have achieved success, although they require substantial computational resources and have certain limitations in real-time processing. In contrast, single-stage algorithms, represented by methods like YOLO (Redmon et al., 2016) and SSD (Liu et al., 2016), offer faster detection speeds but lower accuracy. These models perform effectively on static images and general large-scale datasets but often struggle to identify small targets in cluttered and dynamic environments, particularly due to the information loss associated with small targets. Issues such as motion blur, object occlusion, lighting variations, angle changes, and device defocusing in video object detection highlight the necessity for more efficient and accurate methods for detecting small drones in complex environments (Jiao et al., 2021). To address the above challenges, gaining an understanding of the operational mechanisms of the biological retina (Yücel et al., 2003) offers valuable insights. Serving as the initial stage in visual information processing, the biological retina is responsible for converting optical stimuli into electrical signals. These signals undergo preliminary processing by the retinal neuron network before being transmitted to the output neurons of the retina—ganglion cells. Ultimately, they are transformed into action potentials and conveyed to the visual center via the optic nerve. The biological retina is endowed with highly specialized functions, encompassing high-resolution color perception, swift response to dynamic images, and effective processing of intricate scenes. These attributes equip the retina to manage a wide range of visual information, facilitating complex visual tasks such as motion detection, depth perception, and image segmentation (Neuroscience, 2020).

Despite drawing inspiration from the biological visual system for feature extraction and hierarchical processing, traditional visual perception algorithms struggle to adapt to swift-moving targets or intricate backgrounds, particularly within dynamic environments where erroneous detections are prevalent. Unlike conventional artificial neural networks, the biological retina possesses the ability to directly process dynamic temporal information and adjust to complex environments through mechanisms such as neural plasticity, a trait that proves challenging to completely replicate (Wohrer and Kornprobst, 2009; Hagins, 1972; Field and Chichilnisky, 2007; Beaudot et al., 1993). Bio-inspired models that emulate the workings of the biological retina offer improved capabilities in extracting motion features, thereby elevating the precision and dependability of object detection.

Spiking Neural Networks (SNNs), recognized as the third generation of neural networks (Maass, 1997), are computational models that closely emulate biological neural networks by processing information through the spiking activity of neurons. Unlike conventional Artificial Neural Networks (ANNs), SNN neurons communicate using binary events rather than continuous activation values. This approach not only mirrors the structure and function of the biological retina but also encodes and transmits information through the processing of temporal spike sequences, displaying spatiotemporal dynamic characteristics. This intricate activity pattern enables the system to maintain overall stability while adapting to environmental changes and acquiring new motion information through plasticity mechanisms, mirroring the visual filtering observed in biological systems. Due to their event-driven nature, SNNs can more accurately utilize energy when processing sensor data similar to the retina (Jang et al., 2019), which is particularly useful for drone applications (Dupeyroux et al., 2021; Sanyal et al., 2024). They can be applied to tasks requiring real-time or edge computing and can integrate with neuromorphic processors (Calimera et al., 2013) to achieve rapid response in challenging scenarios. Recent years have witnessed the versatility and efficiency of SNNs across diverse domains (Mehonic et al., 2020; Kim et al., 2020), notably excelling in speech recognition (Wu et al., 2020; Wu et al., 2018), image classification (Kim et al., 2022; Vaila, 2021; Zhu et al., 2024), sensory fusion (Glatz et al., 2019), motion control (Glatz et al., 2019), and optical flow computation (Gehrig et al., 2020; Ponghiran et al., 2022). Compared to earlier methods such as Convolutional Neural Networks (CNNs) and optical flow techniques, SNNs provide a more biologically plausible and energy-efficient solution, particularly well-suited for feature extraction of small drones in scenarios where rapid adaptation to environmental changes is crucial, and facilitates a synergistic balance between the efficient encoding and processing of visual information and biological authenticity.

In time-sensitive scenarios, the incorporation of motion features proves advantageous for visual perception tasks, particularly in the context of processing temporal information and its implications for learning mechanisms. Currently, there is no research utilizing SNNs to model the dynamic visual information processing mechanisms of the retina and apply the motion information extracted by SNNs to drone object detection tasks. To address this issue and achieve both biological realism and efficiency in handling complex dynamic visual tasks, we introduce dynamic temporal information into the retinal output model. We have devised a primary motion saliency estimation algorithm, exclusively comprising an SNN architecture, serving as a visual motion perception model to emulate the processing and output of dynamic information by the biological retina in visual perception tasks. The acquired motion information is subsequently amalgamated with spatial information for utilization in drone object detection tasks.

Our research encompasses several key aspects: First, we develop a magnocellular pathway dataset based on the biological characteristics of the retinal magnocellular pathway computational model. Second, we investigate how SNNs encode and transmit temporal information through spike sequences, emulating the biological retina ex-traction of dynamic visual information. Third, we propose a biologically inspired visual motion perception model, referred to as the Magno-Spiking Neural Network (MG-SNN). This model comprises a computational framework solely using spiking neural networks to process visual information, acting as a primary motion saliency estimation model aligned with the retinal magnocellular pathway. We validate the accuracy of the SNN model in extracting motion features. Finally, the MG-SNN is used as a motion feature extraction module, which is combined with the object detection model to form a target detection framework, and the experimental results indicate that the framework can accurately identify and detect low-altitude drone targets.

Specifically, the main contributions of this paper are summarized as follows:

- This research is the first attempt (to our knowledge) to effectively simulate the magnocellular function of extracting motion features of objects using a two-layer spiking neural network framework, as a motion detection plug-in geared towards object detection;
- Experimental validation shows that the MG-SNN model closely matches biological retina processing and enhances object detection accuracy and reliability, demonstrating the potential of biologically inspired SNN models in drone detection;
- In conjunction with the magnocellular pathway computational model, we design the Visual-Magnocellular Dynamics Dataset (VMD) for supervised learning of motion features. The MG-SNN, combined with popular traditional object detection models, improves small drone detection performance in complex backgrounds.

The remainder of this paper is organized as follows. In Section 2, we introduce the related work on motion saliency computation and motion object detection, biologically inspired retinal models, and spiking neural networks for visual tasks. In Section 3, we present the retinal-inspired spiking neural network module for drone motion extraction and object detection architecture. This includes introducing the magnocellular pathway dataset inspired by the retinal magnocellular pathway computational model, explaining the proposed spike temporal encoding method for processing input video frames, and discussing in detail the primary motion saliency estimation model MG-SNN based on the SNN architecture, along with theoretical derivations and feasibility explanations of the proposed method. In Section 4, we describe the comparative experimental conditions and evaluation methods for motion feature extraction and object detection, followed by a thorough discussion and analysis of the experimental results. Finally, in Section 5, we provide a summary of the entire paper.

## 2 Related work

### 2.1 Motion saliency computation and motion object detection

The initial research into motion saliency calculation initially emphasized single visual cues, such as motion speed or direction. However, these methods often lacked adaptability to rapidly changing scenes. Researchers utilized techniques like Support Vector Machines (SVM) to improve the prediction of salient motion areas, but these approaches incurred substantial computational loads when handling large-scale video data. In recent years, composite models (Wang et al., 2017; Bi et al., 2021) have gained traction by integrating multiple visual cues to enhance overall system performance. Notably, models (Maczyta et al., 2019) have been employed to extract motion saliency over video segments, leveraging their exceptional feature learning capabilities for dynamic scene analysis. Similarly, Guo et al. (2019) calculated motion saliency between adjacent frames by analyzing optical flow fields to obtain foreground priors. They utilized a multi-cue framework to integrate various saliency cues and achieve temporal consistency.

In the early research on moving object detection, traditional algorithms focused on simple techniques such as background subtraction and threshold processing. For instance, reference (Yang et al., 2012) utilized dynamic thresholds to compensate for the shortcomings of fixed-threshold background subtraction, enabling timely background updates and overcoming the limitations of traditional background update methods. While these techniques are straightforward to implement, their performance in dynamic backgrounds is suboptimal and offers limited potential for improvement. With advancements in computational power, methods integrating multiple sensory information (such as motion, color, and geometry) (Bhaskar, 2012; Minaeian et al., 2015) began to be employed to enhance the accuracy and robustness of object detection. Compared to traditional algorithms that detect small target locations through inter-frame target association, deep learning-based methods operate directly on keyframes by generating bounding boxes around targets to detect and track moving objects more effectively in complex environments. For example, methods from the YOLO series (Redmon et al., 2016) and the SSD series (Liu et al., 2016) regress directly on the input image to obtain localization and classification information for motion objects.

The algorithms exhibit certain limitations in the task of motion object detection, potentially resulting in the oversight of smaller or infrequently appearing objects, the loss of temporal information, and insufficient accuracy in dense scenes. In specific situations, they require more computational resources, which may reduce their suitability for highly real-time applications. Our approach departs significantly from previous methodologies by directly integrating biological principles into object motion sensitivity, as opposed to relying on arbitrary network architectures or parametric models. Opting for spiking neural networks over artificial neural networks holds promise in providing a more organic approach to processing visual information, thereby enabling the attainment of detection outcomes that more closely mirror human visual perception.

### 2.2 Biologically-inspired retinal models

The initial research into motion saliency calculation initially emphasized single visual cues, such as motion speed or direction. However, these methods often lacked adaptability to rapidly changing scenes. In the realm of bio-inspired retinal models, initial research centered on emulating the photoreception and primary processing mechanisms characteristic of the human retina. For instance, Melanitis and Nikita, 2019 explored the simulation of photoreceptors and initial signal processing in computational models of the retina, to replicate the early stages of visual processing observed in biological systems. Recently, researchers have been investigating the potential use of these models in more complex visual tasks for feature extraction and decision support. For example, Aboudib et al. (2016) proposed a bio-inspired framework for visual information processing that specifically focuses on modeling bottom-up visual attention, utilizing the retinal model for testing and theoretical validation.

Given the characteristics of various types of neurons and neural circuits in the retina, researchers have developed a range of models tailored to distinct task types within the retina-inspired visual motion perception domain. Most of these models are multi-scale CNNs and Recurrent neural network (RNNs) models, constructed to mimic biological visual perception mechanisms. For instance, Zheng et al., 2021 designed a model comprising feed-forward convolutional filters and recurrent units to represent temporal dynamics displayed in continuous natural videos and neural responses within the retina; McIntosh et al. (2016) developed a deep learning model based on CNNs to capture responses to motion stimuli; Parameshwara (2022) designed a retinal-inspired visual sensor model and framework, integrating CNNs and LSTMs to execute motion perception tasks. Moreover, Lehnert et al. (2019) introduced a retinal-inspired visual module encompassing CNN and LSTM layers for navigation perception tasks in complex settings. These models extract motion features from time-series images to identify and analyze motion stimuli and thus might lose temporal information.

Furthermore, attention mechanisms, inspired by the biological visual system, are used to enhance the recognition accuracy of significant motion targets by emulating the retinal focus mechanism on crucial visual features to detect prominent moving objects within videos. Lukanov et al. (2021) proposed an end-to-end model grounded in feature saliency, influenced by the retinal sampling mechanisms observed in primates; the BIT model (Sokhandan and Monadjemi, 2024) employs a bio-inspired mechanism with an attention mechanism to effectively track targets in video sequences, and Malowany and Guterman (2020) utilize deep feedforward CNNs combined with top-down attention mechanisms from the human visual system for object recognition tasks.

While drawing inspiration from the multilayered visual systems and yielding outputs consistent with visual mechanisms, these models fall short of replicating the information-processing pathways of the human brain. By simplifying complex biological structures and functions to achieve specific capabilities, they do not truly reflect the transmission and processing of information in the temporal dimension.

### 2.3 Spiking neural networks for visual tasks

Furthermore, attention mechanisms, inspired by the biological visual system, are used to enhance the recognition accuracy of significant motion targets by emulating the retinal focus mechanism on crucial visual features to detect prominent moving objects within videos. Initially, the application of SNNs in visual tasks was primarily focused on basic image and video processing tasks, such as image reconstruction and simple object recognition. These tasks utilized the temporal dynamics of SNNs to mimic the primary stages of visual perception. Despite the tremendous success of CNNs in visual tasks, research into SNNs aims to leverage their event-driven characteristics for encoding information, with the expectation of achieving greater efficiency in power consumption and algorithmic complexity. Most relevant to processing visual motion information is the SpikeMS model (Parameshwara et al., 2021), which accurately segments and tracks dynamic moving targets in video sequences. This model uses an architecture that combines multilayer CNNs with SNNs to extract spatial features from video sequences and ultimately produces segmentation results for dynamic targets. Similarly, the Spike-FlowNet model (Lee et al., 2020) utilizes a deep SNN encoder and an ANN decoder architecture for self-supervised optical flow estimation. Additionally, the U-Net-like SNN model (Cuadrado et al., 2023) integrates the U-Net architecture with SNN neuron models to extract motion and optical flow information in the temporal dimension, by combining event-based camera data with SNNs for optical flow and depth prediction. Another architecture (Hagenaars et al., 2021) designed for optical flow estimation processes event data using an ANN-SNN hybrid approach. It is evident that most visual motion perception models related to SNNs are based on hybrid ANN-SNN architectures. Although SNN neurons are introduced to handle the temporal dimension, fully simulating the dynamic behavior of neurons remains challenging, and there is a performance and accuracy loss during the conversion process.

Frame-based images and feature vectors need to be encoded as spike trains to be processed within SNNs. These spike events are non-differentiable, making traditional backpropagation methods challenging to employ. Early attempts at training SNNs focused on biologically inspired Hebbian mechanisms (Sejnowski and Tesauro, 1989). Spike Time Dependent Plasticity (STDP) (Mozafari et al., 2018) strengthens synapses that may aid in neuron firing, thus avoiding the gradient issue. In ANN-SNN methods, input representations are formed by binning events within time intervals and converting them into image-based frame structures, referred to as “event frames.” Most dynamic information processed in SNNs also originates from event data generated by Dynamic Vision Sensors (DVS) (Haessig et al., 2019). However, our method is distinct in that it directly feeds video frames into the network through spike encoding, capitalizing on the temporal properties of SNNs combined with the temporal properties of spike trains. By encoding pixel intensity as spike timing, this approach naturally reduces the processing of redundant information while preserving all significant information, as only notable visual changes trigger spikes.

## 3 Materials and methods

This section will provide a detailed overview of the learning and inference process of the algorithm developed using SNNs, which simulates the retinal channel process of handling motion information and extracting accurate motion feature information. The extracted visual dynamic features are then used as a motion guidance module applied to drone object detection. First, we will introduce the overall architecture of the retinal-inspired spiking neural network for drone motion extraction and object detection. Then, we will describe the two main components: extracting visual motion information with MG-SNN and applying it to drone object detection. In the first part, the MG-SNN (Magno-Spiking Neural Network) model for motion saliency estimation includes the design of the Visual Magnocellular Dynamics Dataset (VMD), inspired by the computational model of the retinal magnocellular pathway. We will discuss the process of handling multiple frames through a spike temporal encoding strategy. Subsequently, we propose a Dynamic Threshold Multi-frame Spike Time Sequence backpropagation method (DT-MSTS) based on dynamic thresholds and the STDP rule to guide the learning of the SNN network. In the second part, concerning the application to object detection, we will primarily discuss combining MG-SNN with the YOLO model to achieve the task of detecting small drone targets.

### 3.1 Overall architecture

**Figure 1:**
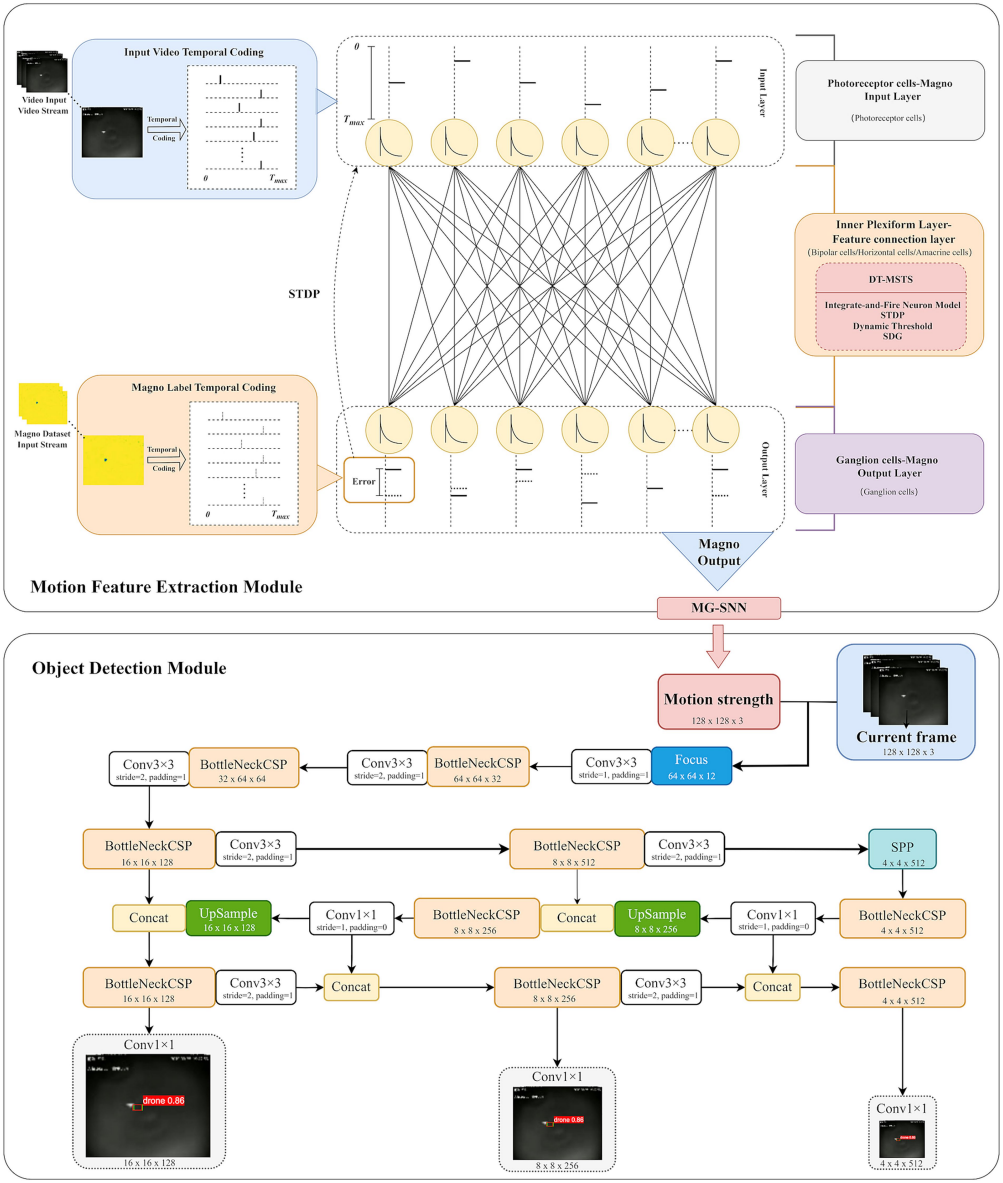
This is the overall architecture of the drone motion extraction and object detection system based on a retina-inspired spiking neural network.

The overall architecture of the drone motion extraction and object detection system based on a retina-inspired spiking neural network is illustrated in Figure 1. In the motion feature extraction module, MG-SNN is constructed by modeling the structure of the biological retina. The input video stream is converted into a temporal spike sequence using a spike temporal encoding strategy in the photoreceptor simulation layer. These sequences then undergo processing in the inner plexiform layer (IPL). During forward propagation in the IPL, the integrate-and-fire (IF) neurons in each layer integrate the presynaptic spike sequences. By employing a dynamic threshold mechanism, thresholds are dynamically calculated based on the time steps of the input spikes, enabling IF neurons to determine whether to fire at specific moments. This allows for synchronous processing of each video frame and the generation of corresponding postsynaptic spike sequences. Simultaneously, the label information of the magnocellular pathway dataset is also converted into spike time sequences using the spike temporal encoding strategy, guiding the subsequent backpropagation process. Ensuring that each neuron can fire at least once, synaptic weights are adjusted according to the spike times of the neurons. This process accurately measures the differences between the output spike sequences and the target spike sequences for supervised learning. It not only simulates the learning process of bipolar cells, horizontal cells, and amacrine cells in the biological retina but also preserves temporal precision.

In the ganglion cell output layer, the output corresponds with motion saliency estimation consistent with the magnocellular pathway. Information is transmitted through discrete-time sequences in the network layers, aligning with the dynamic processing characteristics of the biological retina. After processing the forward and backward propagation of the input spike time sequences within a time step, the membrane potentials of all IF neurons are reset to zero, ensuring stability and continuity when the network processes multiple frames continuously.

In the object detection module, the visual motion features extracted by MG-SNN are combined with the YOLO model (Redmon et al., 2016) to perform drone object detection tasks. This combination enhances the detection capability of small targets in dynamic scenes, achieving accurate detection and rapid response. YOLOv5 is primarily utilized in this experiment. All input channels are first sliced and sent to the convolutional layer, to create a visual object detection model based on SNN motion guidance.

In this implementation, the focus is placed on achieving high detection accuracy in challenging scenarios. MG-SNN serves as a motion-guidance module that extracts dynamic features from video frames and generates a motion intensity map, converting spiking activity into a single-channel grayscale image where dynamic regions are assigned higher intensity values (e.g., 1) and static backgrounds are assigned lower intensity values (e.g., 0). Following the YOLOv5 framework, all input channels are sliced and sent to the convolutional layers. The convolutional responses of the motion intensity map and preprocessed video frames are concatenated and passed into the detection pipeline. This design ensures that regions with higher motion intensity responses are more likely to be activated during subsequent processing, thereby enhancing the detection of dynamic objects within the scene. The synchronized processing of MG-SNN outputs with the original video frames ensures that the entire object detection framework operates in real time without introducing latency. This architecture highlights the flexibility and utility of MG-SNN as a plug-and-play module that enhances object detection tasks. It effectively balances computational efficiency with detection accuracy, addressing the challenges of detecting small and dynamic objects in complex environments.

### 3.2 Drone motion feature extraction based on retinal-inspired spiking neural networks

#### 3.2.1 Magnocellular pathway dataset inspired by the retinal magnocellular pathway computational model

**Figure 2:**
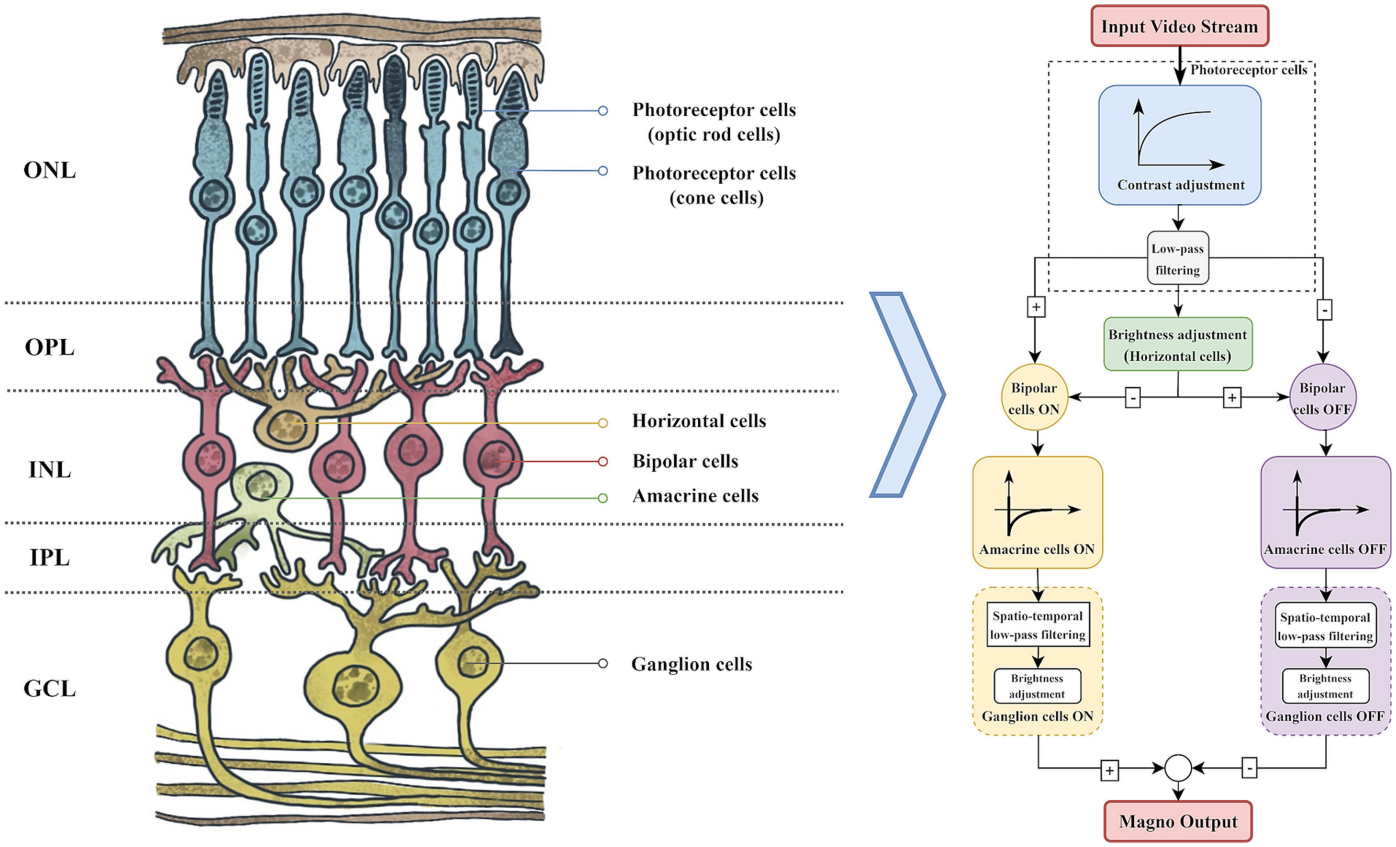
Conversion of the retina to the magnocellular pathway computational model.

The structure and function of the retina (Yücel et al., 2003) are the cornerstone of the visual system, with its layered structure facilitating the efficient transmission and processing of visual signals. As illustrated in Figure 2, these layers consist of the Outer Nuclear Layer (ONL), Outer Plexiform Layer (OPL), Inner Nuclear Layer (INL), Inner Plexiform Layer (IPL), and Ganglion Cell Layer (GCL). The ONL houses photoreceptor cell bodies, while the OPL and IPL serve as synaptic connection layers. The INL includes horizontal, bipolar, and amacrine cells (Stacy and Lun Wong, 2003). Horizontal cells regulate the electrical signals of photoreceptor cells through lateral inhibition, while amacrine cells are responsible for signal processing within the retina by forming synapses with ganglion and bipolar cells. Ultimately, the processed visual information is conveyed to the primary visual cortex in the form of action potentials (Arendt, 2003; Benoit et al., 2010).

The biological visual system processes visual information through two parallel pathways, one dedicated to motion information and the other to static appearance information (Bock and Goode, 2008). These pathways are commonly referred to as the magnocellular path-way (Magno) and the parvocellular pathway (Parvo). The magnocellular pathway plays a crucial role in the processing of visual motion information.

We referred to the magnocellular pathway computational model proposed by Benoit et al. (2010). According to this model, video streams are processed through photoreceptor cells to acquire visual data and normalize light intensity (Beaudot, 1994), thereby enhancing the contrast in dark areas of the video frames while maintaining the visibility of bright areas. The processed frames undergo low-pass filtering and pass through the ON/OFF channels of horizontal and bipolar cells, forming synaptic triads. In the magnocellular pathway, amacrine cells establish connections with bipolar cells and ganglion cells, thereby providing high-pass filter functionality that enhances sensitivity to temporal and spatial changes in visual information. When processing spatial information, ganglion cells act as spatial low-pass filters and compress the contrast of video frames, thereby enhancing low-frequency spatial motion contour information. This dual functionality allows ganglion cells to play a crucial role in integrating visual information, particularly in visual tracking and target recognition in dynamic environments.

**Figure 3:**
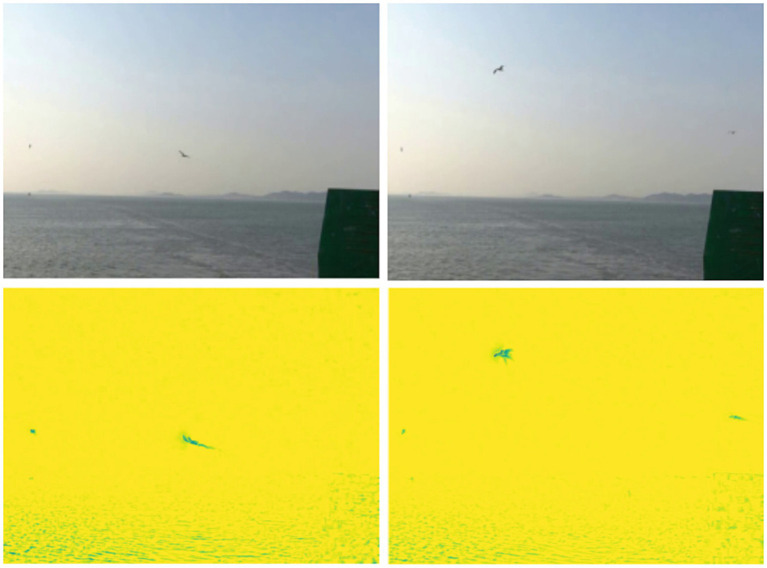
Input video images of flying birds with magnocellular pathway outputs, respectively, are the results of the two frames before and after. In order to obtain better viewing results, we performed min-max normalization of the dynamic motion.

By processing multi-frame historical information, it demonstrates exceptional sensitivity to moving objects, effectively filtering out noise and static backgrounds, as illustrated in Figure 3. This capability is crucial for extracting motion intensity information from visual scenes, facilitating attention guidance and target search. Leveraging this motion processing response, the magnocellular pathway improves focus on potential target areas while adeptly suppressing static backgrounds, which is particularly valuable in visual information processing.

**Figure 4:**
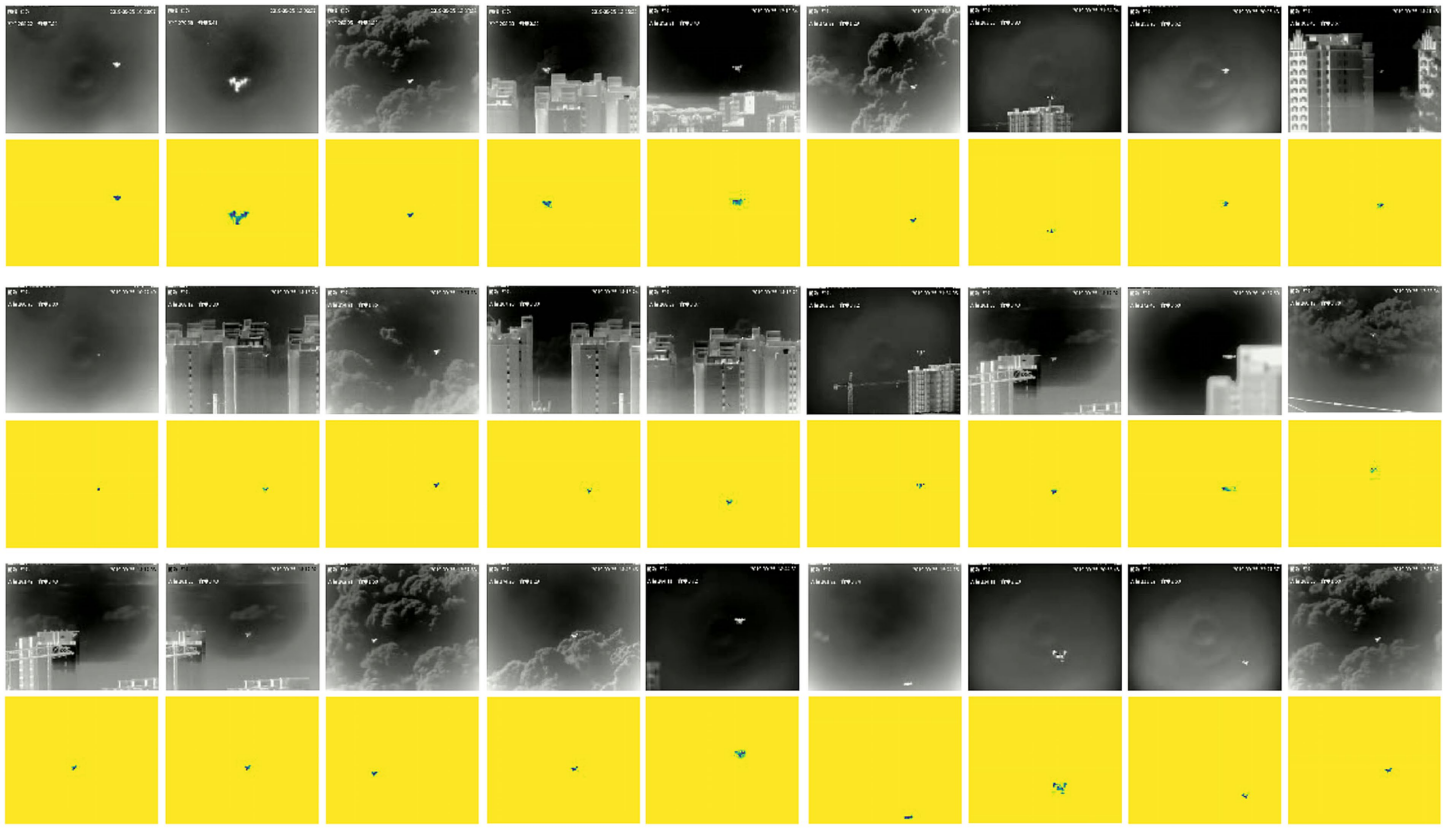
The Anti-UAV2021 challenge dataset and VMD dataset contain large and small objects on clear backgrounds, as well as complex backgrounds (clouds and cities).

In this research, we utilized the output from the magnocellular pathway as the label information for our neural network model and developed the Visual Magnocellular Dynamics Dataset (VMD) as illustrated in Figure 4. This dataset is constructed based on the Anti-UAV-2021 Challenge dataset^1^ and the Anti-UAV-2023 Challenge dataset (Zhao et al., 2023). The videos showcase natural and man-made elements in the backgrounds, such as clouds, buildings, trees, and mountains, realistically simulating scenarios encountered in drone surveillance tasks, the dataset includes target objects of varying sizes, from large to extremely small, intensifying the difficulty of object detection. Furthermore, the Anti-UAV-2023 Challenge dataset to enrich the VMD dataset, aimed specifically at small target recognition tasks, which include more challenging video sequences featuring dynamic backgrounds, complex rapid movements, and small targets, thereby encompassing a wider range of small target drone scenarios.

The VMD dataset comprises a total of 650 video samples, divided into 500 training samples and 150 test samples, each showcasing various natural and man-made diverse scenes with target objects of small sizes, scenes such as open skies, urban environments, forested areas, and mountainous regions. Motion complexity is introduced with sequences containing both static and dynamic backgrounds, and targets moving at different speeds and directions, challenging the motion detection capabilities of the model. The VMD dataset is created based on the magnocellular pathway computational model and is developed using the bioinspired library in OpenCV. Several preprocessing steps are applied to ensure the quality and consistency of the dataset: normalization of pixel values, setting the video frame resolution to 120 × 100 pixels to ensure computational efficiency, and adjusting each video segment to a frame rate of 20 frames per second with durations ranging from approximately 5 to 10 s, and only the content within the salient bounding boxes is retained to ensure precise labeling. The choice of a 120 × 100 resolution is a practical balance that provides sufficient detail for detecting and identifying small drone targets in complex scenarios. Compared to the simpler tasks often addressed by existing models, such as MNIST handwritten digit classification (32 × 32 resolution), our approach processes more complex inputs while maintaining an efficient computational profile. This resolution ensures that the detection framework operates effectively without compromising the precision required for small drone detection.

#### 3.2.2 Video frame processing based on spike temporal encoding

To replicate visual processing akin to that of the human brain and extract motion features, it is crucial to effectively retain and transform the wealth of information present in external stimuli into sequences of neuronal action potentials. The selection of an appropriate encoding strategy is vital for connecting neuronal action potential sequences with behavioral information and for closely integrating the mechanisms of processing in the primary visual cortex with spiking neural networks (Field and Chichilnisky, 2007). Currently, two main types of spike encoding are employed in SNNs (Brette, 2015) rate coding and temporal coding.

In most sensory systems, neurons adjust their firing frequency according to the frequency and intensity of stimuli. However, rate coding (Field and Chichilnisky, 2007; Salinas et al., 2000) does not fully account for the rapid response capability of the visual system. Furthermore, accurately representing complex values with single neuron spikes is challenging and results in a loss of temporal information. Visual information transmission involves multiple synaptic transmissions, with each processing stage being extremely brief. Consequently, the firing frequency of neurons in the primary visual cortex is relatively low in these rapid response processes, a single neuron may only fire 0 or 1 action potential, making it impossible to estimate instantaneous firing rates based on the interval between two action potentials (Thorpe et al., 2001; Salinas et al., 2000), the use of changes in firing frequency to encode specific features of complex stimuli is inadequate.

To simulate the flexibility and adaptability demonstrated by the primary visual cortex in processing images or video data, this study adopts a temporal encoding strategy for spike encoding of input information. By representing specific values at precise moments of a single spike, the temporal structure of action potential sequences can encode information related to temporal changes in stimuli (Bair and Koch, 1996), such as the temporal variations in stimulus intensity. This allows for a more accurate representation of input grayscale value information in the temporal dimension.


(1) $$T_{i}=\left(1.0-I_{i}\right)\times T_{\text{max}}$$

**Figure 5:**
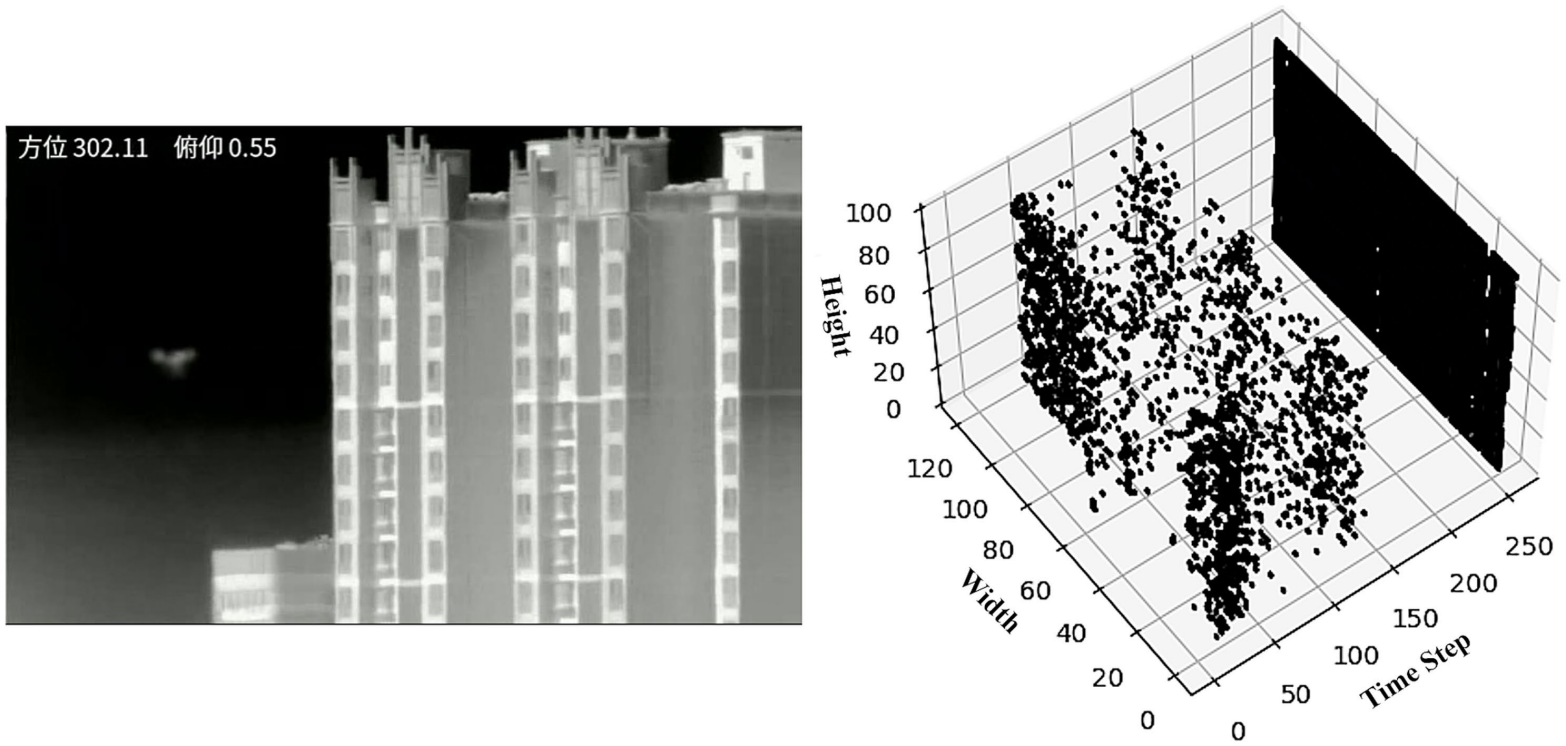
The visualization depicts a single drone video frame along with its spike temporal coding sequence spanning 256-time steps.

In subsequent experiments, we determine the activation time $T_{i}$ of each input neuron based on the normalized intensity value of the i -th pixel as shown in Equation 1. For this purpose, we employ a spike temporal encoding method to process the input video frames. The specific encoding formula is described as follows:

$T_{\text{max}}$ represents the maximum time step of the input spike sequence, while $I_{i}$ is the normalized intensity value of the i -th pixel. Under this encoding mechanism, each pixel in the input layer generates a single spike at a specific moment $T_{i}$ , forming a temporal spike sequence. The higher the intensity value of the input, the earlier the corresponding spike firing time. Figure 5 illustrates the visualization result after encoding a video frame.

Temporal encoding utilizes earlier spike firing times to represent pixels with higher grayscale values, while higher thresholds cause neuronal discharge to delay, indicating that the pixel has a lower grayscale value. Our experiments make use of this time encoding mechanism to accurately map the temporal dimension of visual information, enabling efficient and sensitive processing of visual stimuli within the spiking neural network.

#### 3.2.3 Spiking neurons

In this study, we utilized Integrate-and-Fire (IF) neurons to develop a motion saliency estimation model using a pure SNN architecture. IF neurons operate by accumulating incoming signals until a certain threshold is reached, after which an action potential or “spike” is generated, and then resets its state, mimicking the basic behavior of biological neurons (Smith et al., 2000).

**Figure 6:**
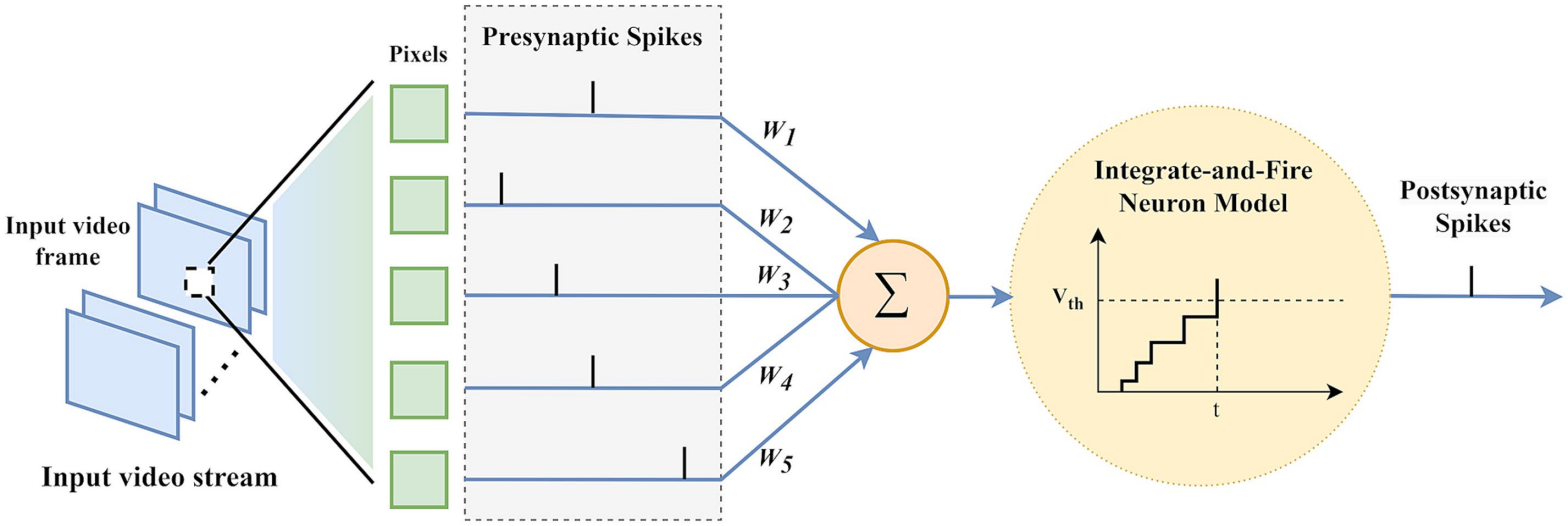
Processing of multi-frame video frames by IF neurons.

As Figure 6 illustrates, input video frames are encoded into a time spike sequence of length [0, $t_{\text{max}}$ ], then the presynaptic spike sequence enters the network. Through IF neurons, when the membrane potential of an IF neuron exceeds the threshold potential $V_{th}$ , it generates a postsynaptic spike sequence. To ensure that non-firing neurons also transmit video frame information, it is defined that non-firing neurons emit a single spike containing minimal information at time $t_{\text{max}}$ . The simplified formula for IF neurons is shown in Equation 2:


(2) $$\begin{array}{l} \left(t\right)=V\left(t-T\right)+\underset{l}{\sum }w^{l}\left(t\right)S^{l-1}\left(t\right)\;\;\\ \mathrm{if}\;V\left(t\right)\geq V_{th}\;\mathrm{then}\;r\left(t\right)=1,V\left(t\right)=V_{\mathrm{reset}} \end{array}$$

where $V\left(t\right)$ represents the membrane potential of the neuron, $w^{l}$ is the synaptic weight between layers, and $S^{l-1}\left(t\right)$ is the incoming spike sequence from the previous layer. The spike firing rate $r\left(t\right)$ is 1 when a spike is emitted and 0 when no spike is emitted. When the membrane potential exceeds the threshold $V_{th}$ , the membrane potential is immediately reset to the resting potential $V_{\mathrm{reset}}$ , which is typically set to 0.

During the processing of video frames after spike temporal coding by IF neurons, the threshold of the neuron affects its moment of discharge and maintains the system homeostasis (Abbott, 1999). Based on the leaky adaptative threshold (LAT) mechanism (Falez, 2019), this study introduces a dynamic threshold mechanism that linearly increases with the input time steps. This mechanism is the first attempt to combine dynamic threshold adjustment with a video frame time encoding strategy, aiming to emphasize the importance of spike information in early time steps, which are considered to contain more distinct features compared to later information. This strategy, by enhancing the sensitivity of neurons to high-intensity inputs in early time steps, optimizes the efficiency of information processing.

The dynamic threshold mechanism contains a baseline threshold $V_{th}$ , which is linearly with the increase in input time steps to preserve the unique response characteristics of each IF neuron as in Equation 3. The threshold adjustment $D_{th}$ occurs when the neuron is excited and upon receiving inhibitory spikes, reducing the discrepancy between the actual firing time $T_{i}$ and the expected firing time $T_{\mathrm{label}}$ . The introduction of a dynamic threshold allows the neuron threshold to adjust automatically, encouraging the firing time $T_{i}$ to approach the target time $T_{\mathrm{label}}$ , while maintaining the system equilibrium. The threshold learning rate θ allows for adjustment of the rate of threshold change based on specific circumstances. The specific adjustment rule is as follows:


(3) $$D_{th}=V_{th}+\theta T_{i}$$

This rule is designed to correct the timing error between the actual firing timestamp $T_{i}$ and the target timestamp $T_{\mathrm{label}}$ each time the neuron fires. The specific value of the threshold learning rate θ depends on the dataset and characteristics of the input video frames and requires an exhaustive search within the range [0, $t_{\text{max}}$ ] to determine the optimal value. Since the membrane potential is determined by synaptic weights and the input spike sequence, designing an appropriate dynamic threshold rule can effectively enhance the influence of the input spike sequence on the membrane potential, thereby significantly improving the overall performance of the network.

#### 3.2.4 Backpropagation method

The Spike-Timing Dependent Plasticity (STDP) rule adjusts synaptic strengths based on the precise timing of neuronal spikes. This rule leverages the temporal relationships between neuron firing times (Diehl and Cook, 2015), not only effectively encoding temporal information within neural circuits but also facilitating the update of specific synaptic weights. By adjusting synaptic weights based on the relative timing differences between input and output spikes, a biologically plausible learning mechanism is achieved.


(4) $$V_{\mathrm{total}}\left(t\right)=\underset{t^{l}\in \left\{r\left(t\right)=1\right\}}{\sum }V\left(t^{l}\right)$$

This study introduces a method that combines the STDP rule (Bi and Poo, 1998; Caporale and Dan, 2008) and a time error function—Dynamic Threshold Multi-frame Spike-Time Sequence Backpropagation Method (DT-MSTS)—to perform backpropagation computations after temporal encoding of video frames. Our approach made improvements based on the BP-STDP method described in the literature (Sjöström and Gerstner, 2010). As shown in Equation 4, an IF neuron with no leak characteristics accumulates membrane potential over time with the output spike sequence and fires when it’s membrane potential $V\left(t\right)$ reaches the neuron threshold $V_{th}$ .

Considering that network decisions rely on the first spike signal from the output layer, earlier spikes thus contain more information. Under the same input and synaptic weights, the membrane potential of an IF neuron approximates the activation value of the Rectified Linear Unit (ReLU) neuron Tavanaei and Maida, 2019 as in Equation 5. We can assume there is an approximate relationship between the output of the ReLU neuron and the firing time $t^{l}$ of the corresponding IF neuron:


(5) $$y^{l}\sim t_{\text{max}}-t^{l}$$

In the forward propagation process we constructed, $y_{j}^{l}$ represents the activation value of the j -th neuron in layer l , and $z_{j}^{l}$ is the weighted input of that neuron. As IF neurons approximate ReLU neurons, in the ReLU function, $\partial y_{j}^{l}/\partial z_{j}^{l}$ acts as the derivative at that point as shown in Equation 6:


(6) $$\frac{\partial y_{j}^{l}}{\partial z_{j}^{l}}=\{\begin{array}{c} 1\;\mathrm{if}\;y_{j}^{l}>0\;\\ 0\;\mathrm{otherwise} \end{array}$$

However, in IF neurons, since $t_{j}^{l}$ is not a function of $w_{j}^{l}$ , we cannot compute $\partial t_{j}^{l}/\partial w_{j}^{l}$ . For each neuron j in layer l , if its threshold time $t_{j}^{l}$ is less than $t_{\text{max}}$ , the derivative of its membrane potential $V_{j}^{l}$ with respect to input weight $w_{ji}^{l}$ can be calculated through the spike activity of the preceding layer neuron i . If $t_{j}^{l}<t_{\text{max}}$ , then assume $\partial t_{j}^{l}/\partial V_{j}^{l}=-1$ , where $t_{\text{max}}$ is the maximum spike firing time.


(7) $$e_{ij}=\mu {\left(T_{l}^{l}-T_{o}^{l}\right)}^{2}/t_{\text{max}}^{2}$$

If IF neurons fire within the maximum time window, the time error gradient related to the neuron firing time can be calculated. During the learning process, we initially utilize the Stochastic Gradient Descent (SGD) algorithm in conjunction with the backpropagation algorithm to minimize the mean squared time error function. For each training sample, the mean squared time error function $e_{ij}$ is defined as follows in Equation 7:

where $T_{l}^{l}$ represents the target spike firing time, and $T_{o}^{l}$ is the spike firing time output for each layer, μ used for error updating. As the equation illustrates, we introduce the STDP factor $\varepsilon_{i}\left(t\right)$ to guide the backpropagation update process of the time error function. This means that if the firing time of the label information in the magnocellular pathway is earlier than the output spike firing time, synaptic connections will be weakened through a negative feedback STDP factor ( $\varepsilon_{i}\left(t\right)=-1$ ); conversely, if the firing time is later, connections will be strengthened through a positive feedback STDP factor ( $\varepsilon_{i}\left(t\right)=1$ ).


(8) $$\varepsilon_{i}\left(t\right)=\{\begin{array}{c} 1\\ -1\\ 0 \end{array}\begin{array}{c} T_{l}^{l}>T_{o}^{l},T_{l}^{l}\neq t_{\text{max}},T_{o}^{l}\neq t_{\text{max}}\\ T_{l}^{l}<T_{o}^{l},T_{l}^{l}\neq t_{\text{max}},T_{o}^{l}\neq t_{\text{max}}\\ \mathrm{otherwise} \end{array}$$

Thus, combining the mean squared time error function $e_{ij}$ , the total loss function L is defined as:


(9) $$L=\frac{1}{2}\sum_{j=1}^{0}\frac{\mu {\left(T_{j}^{i}-T_{j}^{l}\right)}^{2}}{t_{\text{max}}^{2}}$$

where O is the number of output layer neurons, where μ is time error update parameter. For the output layer $\left(l=o\right)$ , the error term is calculated as:


(10) $$\delta_{j}^{w}=-\frac{\mu \left(T_{j}^{w}-T_{j}^{\alpha }\right)}{t_{\text{max}}^{2}}$$

where $\delta_{j}^{w}$ directly reflects the prediction error of each output neuron. Finally, the error term obtained can be used to adjust the network weights:


(11) $$w_{ji}^{l}=w_{ji}^{l}+\beta \delta_{j}^{w}S_{i}^{l-1}\varepsilon_{i}\left(t\right)$$

where β is the learning rate, which controls the step size of weight updates, and $S_{i}^{l-1}$ is the output of the previous layer of neurons. This ensures that the network can learn to reduce output errors, thereby improving the accuracy of outputs corresponding to the magnocellular pathway according to the calculations in Equations 8–11. Ultimately, the output layer of ganglion cells will produce a time spike sequence that corresponds to that of the magnocellular pathway in the primary visual.

### 3.3 Drone object detection based on retinal-inspired spiking neural networks

The YOLOv5 structure comprises several crucial components that ensure its efficiency and accuracy in object detection tasks. Firstly, YOLOv5 employs the Cross-Stage Partial Network (CSPNet) (Wang et al., 2020) as part of its backbone network, enhancing the model learning capability and generalization ability. CSPNet reduces computational cost while preserving spatial feature information by dividing the feature map into two parts: one that passes directly through dense blocks and another that merges with the backbone network. Additionally, YOLOv5 incorporates the Path Aggregation Network (PANet) (Liu et al., 2018) and the Spatial Pyramid Pooling (SPP) (He et al., 2015) module. PANet enhances feature fusion by combining high-level and low-level features, thereby improving object detection performance. The SPP module acts as a spatial pyramid pooling module, integrating information at different scales through pooling operations of various sizes, effectively expanding the receptive field and capturing more contextual information, which enhances the accuracy of drone detection.

The YOLOv5 structure incorporates multiple convolutional layers, pooling layers, and activation function layers, which collectively enable the model to extract crucial features from images and map these features to specific detection results through the final output layer. The Feature Pyramid Network (FPN) (Lin et al., 2017) connects up sampled mappings with corresponding feature mappings in the down sampling branch.

By integrating the dynamic visual features extracted by MG-SNN as a motion-guidance module with the spatial information present in drone video frames into the YOLOv5 model, the primary motion saliency estimation features output by MG-SNN are linked with the convolutional responses of preprocessed video frames. This connection ensures that regions with higher motion intensity response values are more likely to be activated during subsequent processing. Consequently, YOLOv5 is effectively guided to focus on key dynamic areas during detection, which leads to a reduction in false positives and an improvement in recognition accuracy. This innovative approach realizes the integration of SNNs as a visual motion information guidance module with the spatial appearance information represented by deep neural networks for object detection and recognition.

## 4 Results

In this section, we conduct experiments on the VMD datasets to validate the performance of our model and evaluate its performance across various scenarios. Additionally, we introduced new comparative methods for experimentation and examined the superiority of our model compared to traditional methods based on the magnocellular pathway.

The experiments were conducted on an Ubuntu operating system. The experimental setup was executed on a PC equipped with an AMD EPYC 7502 32-core processor and an A100-PCI-E-40GB GPU. We set the number of training epochs to 20 and employed a learning rate strategy, while the input size for the network was fixed at 120 × 100. The other parameter settings are shown in Table 1.

**Table 1:** This is a table of the parameter settings for MG-SNN.

Parameters	Description	Value
$t_{\text{max}}$	Maximum time step of the input spike sequence	256
$V_{th}$	Baseline threshold for dynamic threshold	0 mV
$V_{\mathrm{reset}}$	Resting potential	0 mV
θ	Dynamic threshold learning rate	0.5
μ	Time error update parameter	0.02
I	Number of input neurons in the photoreceptor layer	12,000
O	Number of output neurons in the ganglion cell layer	12,000
β	Learning rate	10^−6^
Gray level	Maximum gray value of temporal coding video frame	255
$w_{\text{max}}^{o}$	Initialize maximum synaptic weights	1
$w_{\text{min}}^{o}$	Initialize minimum synaptic weights	-1

### 4.1 Quantitative results of motion feature extraction


(12) $$MAE=\frac{1}{N}\overset{N}{\underset{i=1}{\sum }}|T_{i}-\hat{T_{i}}|$$

To quantify and evaluate the performance of MG-SNN in simulating the output of the magnocellular pathway, we employed a statistical error measurement method: Mean Absolute Error (MAE). As shown in Equation 12, for each neuron i in the video frame, where $T_{i}$ is the firing time of the spike sequence output by the i -th neuron in the magnocellular pathway, and $\hat{T_{i}}$ is the firing time of the spike sequence output by the i -th neuron in the MG-SNN, with n being the total number of input neurons.

When the MAE value is smaller, it indicates better model performance. By synthesizing this metric, we can comprehensively assess the model performance on the magnocellular pathway dataset, ensuring the model not only achieves outputs consistent with the visual pathway but also possesses robustness against outliers.

**Figure 7:**
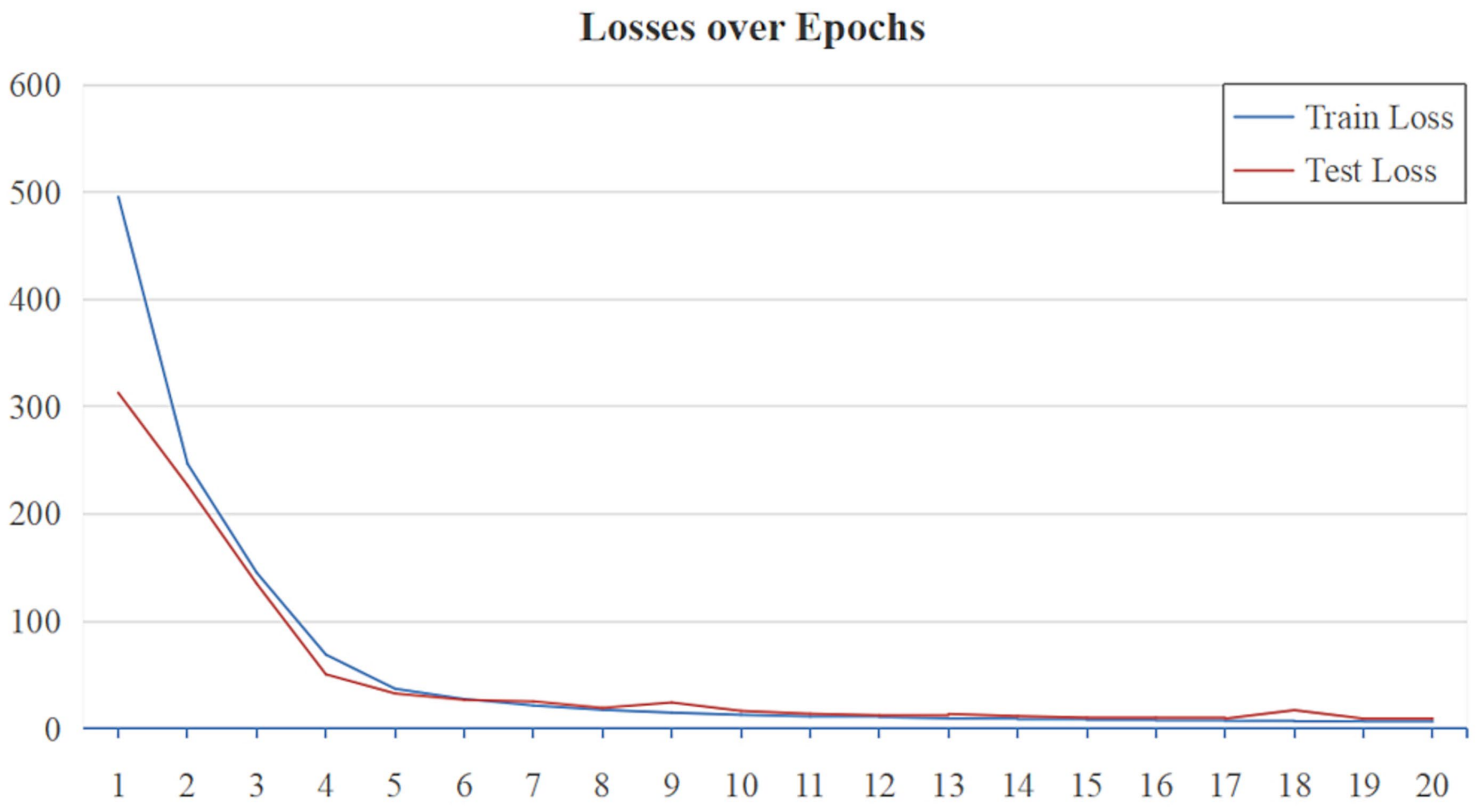
MAE loss curves of MG-SNN on the VMD dataset.

As shown in Figure 7, the performance of MG-SNN is evaluated by analyzing the training and testing MAE loss over 20 epochs on the VMD dataset. Initially, both training and testing losses exhibit a sharp decline, indicating that the model learns and fits the data quickly during the early epochs, the losses converge rapidly, stabilizing after approximately 10 epochs, which illustrates that MG-SNN avoids overfitting, demonstrates a certain level of generalization, and maintains stable learning of spatiotemporal information present in dynamic data.

Visual representations observed in the early visual areas of the primate brain show similarities to those in CNN frameworks trained on real images (Arulkumaran et al., 2017). This indicates that CNN frameworks also possess a degree of brain inspiration, capable of mimicking the hierarchical structure of simple and complex cells, thereby simulating the function of the retina in object perception to provide stable object representations. To enrich the comparative experiments based on this theoretical foundation, we designed a convolutional neural network model with 3 × 3 two-dimensional convolution kernels (referred to as RetinaCNN) to simulate the output of the magnocellular pathway. The structure is 1C16-3C32-3C1. RetinaCNN directly processes grayscale intensity information in the video stream, sequentially through convolution and activation functions in each layer, ultimately generating an output consistent with the magnocellular pathway. Additionally, based on the RetinaCNN model, a spike-time encoding-based CNN-SNN motion saliency estimation model, named RetinaSNN, was developed by replacing the original activation functions with IF neurons. The structure of RetinaSNN is 1C16-IFNode-32C3-IFNode-1C3.

We conducted tests on the VMD dataset, where each network input consists of three video frames. This comparative experiment includes the output results of the magnocellular pathway computational model, the CNN model (RetinaCNN), and the CNN-SNN hybrid model (RetinaSNN). Furthermore, ordinary frame difference (OFD) and multi-frame difference (MFD) methods were added to enrich the comparative experiments. To achieve a processing mechanism consistent with MG-SNN, the multi-frame difference method accumulates data from three frames in the channel dimension for learning.

### 4.2 Qualitative results of motion feature extraction

**Figure 8:**
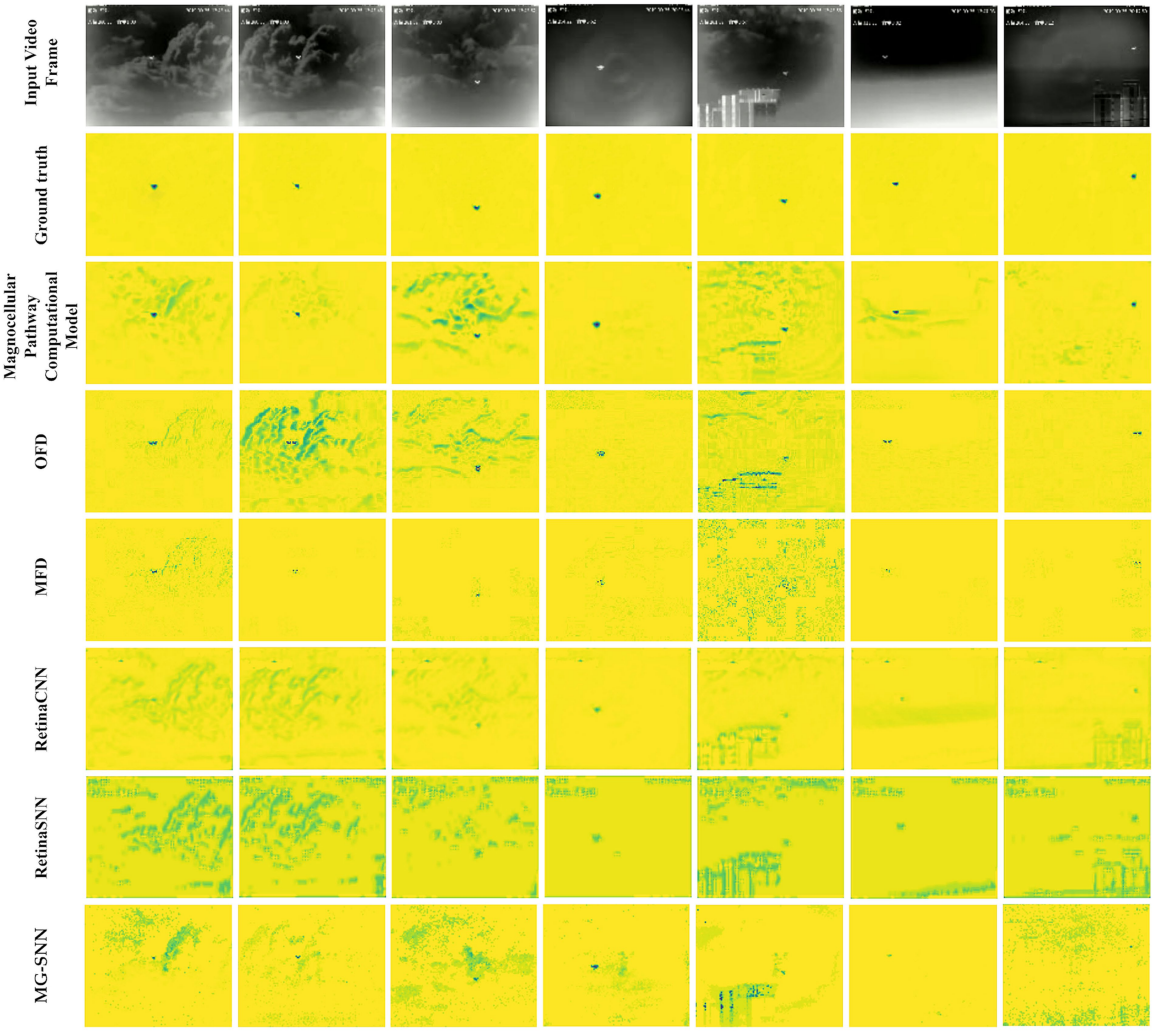
Output results after testing on the VMD dataset. (The first row is the original image; the second row is the VMD dataset labels; the third row is the output of the magnocellular pathway computational model; the fourth row is the output of the ordinary frame difference method (OFD); the fifth row is the output of the multi-frame difference method (MFD); the sixth row is the output of RetinaCNN; the seventh row is the output of RetinaSNN; the eighth row is the output of MG-SNN. The first column shows the results of a small target moving quickly to the right against a cloud background; the second column shows the results of a small target moving quickly to the left against a cloud background; the third column shows the results of a small target moving quickly up and down against a cloud background; the fourth column shows the results of a small target tested against an exposure background; the fifth column shows the results of a small target against a composite background (clouds and city); the sixth column shows the results of a tiny object moving quickly against a cloud background; the seventh column shows the results of a tiny object moving quickly against a city background.).

Since the output of MG-SNN is a temporal spike sequence, for a more stable and accurate analysis of experimental results at the beginning of the test set, it is necessary to transcode the output before performing visual analysis. The visualization results are shown in Figure 8.

The results indicate that most “edge glow” and “video subtitles” phenomena caused by the camera are effectively filtered out regardless of the target size, but MG-SNN does not eliminate all noise interference, as some irrelevant neurons fire prematurely. This does not affect the identification of the main dynamic targets. In complex background test scenarios, compared to metrics such as OFD and MFD, MG-SNN can better focus on filtering and identifying dynamic information, effectively filtering out most of the interference caused by camera shake and moving cloud backgrounds. Its performance falls short in urban backgrounds, possibly due to inadequate filtering of the complex background and the generation of leading spikes by buildings after spike temporal encoding, making it difficult to identify objects clearly. Nonetheless, the neurons corresponding to tiny targets in complex mixed backgrounds can still produce leading spikes, ensuring effective recognition of moving targets.

Table 2 presents the experimental results, showcasing the performance and effectiveness of different methods on the VMD dataset in handling visual perception tasks. In terms of Mean Absolute Error (MAE) performance, MG-SNN demonstrates an ability to achieve an MAE of 6.4733 within a relatively short training period (20 epochs), showcasing its rapid adaptation to initial training data and its quick attainment of optimal performance in the short term. Notably, MG-SNN outperforms traditional 2D convolutional neural networks (RetinaCNN) and hybrid CNN-SNN architectures (RetinaSNN) in terms of accuracy. This superior performance indicates it effectively captures and processes spatiotemporal information. RetinaCNN struggles to process complex dynamic scenes in comparison due to their inadequate capture of deep spatiotemporal features. Furthermore, the lower MAE observed in the CNN-SNN hybrid architecture compared to traditional CNNs indicates that spike-time encoding-based methods can better extract spatiotemporal information to some extent.

**Table 2:** Comparison of experimental results.

Method	Structure	Network structure	Learning	Minimum MAE during the first 20 Epochs	MAE	Epoch
MG-SNN	SNN	12000FC-IFNode-12000FC-IFNode	DT-MSTS (Dynamic thresholds +STDP)	6.4733	6.4733	20
RetinaCNN	CNN	1C16-3C32-3C1	Backpropagation	35.7711	13.7086	200
RetinaSNN	Spiking CNN	1C16-IFNode-32C3-IFNode-1C3	ANN-SNN Conversion	8.8214	5.1386	200

### 4.3 Quantitative results of object detection

In this section, we leverage the motion features generated by MG-SNN for drone object detection. We use the Average Precision (AP) value as a quantitative measure, reflecting the model detection accuracy at varying thresholds. As shown in Equations 13, 14, Precision represents the proportion of correctly detected results, while Recall represents the proportion of all objects that are correctly detected.


(13) $$\mathrm{Precision}=\frac{TP}{TP+FP}$$

(14) $$\mathrm{Recall}=\frac{TP}{TP+FN}$$

TP denotes the number of correctly detected objects, FP denotes the number of non-object targets detected as objects, and FN denotes the number of missed object targets.

In this comparative experiment, MG-SNN utilizes the VMD dataset, consistent with previous experiments, while other models use the Anti-UAV-2021 Challenge dataset and the Anti-UAV-2023 Challenge dataset. All models are provided with identical inputs, which include complex backgrounds such as clouds and buildings, reflecting real-world scenarios in drone surveillance. Due to the limited computational capacity of MG-SNN, the input size is restricted to 100×120. Therefore, the input features were adjusted to 100×120 before feature fusion to obtain the corresponding quantitative results. The Intersection-over-Union (IoU) threshold greater than 0.25 method was employed. Since the input size is small and the images are of low resolution with fewer pixels per target, a higher IoU threshold might cause valid detections to be overlooked. Using an IoU of 0.25, the model achieves a better balance on the 120×100 input images, striking an optimal balance between precision and recall.

#### 4.3.1 Cooperate with different object detection models

We demonstrate the compatibility of MG-SNN with various object detection models by integrating the motion features extracted by MG-SNN with YOLOv6-l (Li et al., 2022), YOLOv5-s, and YOLOv5-x. We also compare the performance of these combined models with the original YOLOv6 and YOLOv5 structures to highlight the superiority of adding motion information. YOLOv6-l decouples the detection head and redesigns it with an efficient decoupled head, enhancing the model’s detection accuracy and convergence speed.

The evaluation results are shown in Table 3. The combination of MG-SNN and YOLO models consistently outperforms standalone YOLO models in terms of detection AP, precision, and recall. Notably, the combination of MG-SNN and YOLOv5-x achieves a precision of 98.3%. Our method improves precision, indicating that it can detect more true objects in complex backgrounds. Further analysis of recall and AP shows that MG-SNN + YOLOv5-x achieves a recall of 81.1% and an AP of 86.1%, both of which are the highest values in Table 3. This demonstrates that the combination not only effectively reduces false positives but also accurately identifies all true targets. The YOLO methods are limited to handling single-frame information, neglecting the processing of motion information in multi-frame inputs. Adding MG-SNN enhances the capability to capture deep spatiotemporal features, resulting in a significant 2.0 to 5.0 AP improvement in the performance of popular object detection algorithms. This improvement indicates that input from MG-SNN effectively compensates for the lack of motion information when dealing with complex dynamic scenes.

**Table 3:** Ablation study on the generalization of MG-SNN when applying to popular object detection methods.

Methods	Precision (%)	Recall (%)	AP (%)
YOLOv6-l	90.6	78.0	82.4
MG-SNN+ YOLOv6-l	**95.6**	**80.6**	**85.0**
YOLOv5-s	93.5	79.2	81.9
MG-SNN+ YOLOv5-s	**95.5**	**79.4**	**84.3**
YOLOv5-x	95.4	77.4	82.8
MG-SNN+ YOLOv5-x	**98.3**	**81.1**	**86.1**

Bold values indicate the best performance for each metric within the respective method comparison.

Table 4 shows that integrating MG-SNN into existing object detection models introduces minimal computational overhead while maintaining real-time performance. For example, after adding MG-SNN, the GFLOPs of the model slightly increase from 203.8 to 204.5, while the FPS improves from 64.5 to 69.9. This demonstrates that the enhanced motion-guidance functionality provided by MG-SNN does not compromise the efficiency of the framework. Specifically, our method is capable of processing up to 69.9 frames per second, making it highly suitable for real-time small drone detection tasks, which effectively balances computational complexity and performance, meeting the demands of dynamic and time-sensitive environments.

**Table 4:** Computational complexity and performance study of MG-SNN when applied to common object detection methods.

Methods	Prrams (M)	GFLOPs	FPS
YOLOv6-l	59.54	150.5	47.9
MG-SNN+ YOLOv6-l	59.54	152.0	45.2
YOLOv5-s	7.01	15.8	128.2
MG-SNN+ YOLOv5-s	7.01	16.0	126.0
YOLOv5-x	86.17	203.8	64.5
MG-SNN+ YOLOv5-x	86.18	204.5	69.9

#### 4.3.2 Comparison to the advanced competing methods

We compared our method with popular object detection algorithms, including YOLOv7 (Wang et al., 2023), CFINet (Yuan et al., 2023), and DyHead (Dai et al., 2021). Compared to YOLOv5 and YOLOv6, the YOLOv7 model introduces a new set of trainable Bag-of-Freebies strategies to enhance detection performance in small targets and complex scenes by better leveraging cross-layer feature fusion. CFINet is a network architecture that improves small object detection performance through coarse-to-fine region proposal networks (RPN) and imitation learning (Yuan et al., 2023). DyHead employs an attention mechanism to unify different detection heads into a dynamic head framework (Dai et al., 2021).

The evaluation results are shown in Table 5. Although YOLOv7x achieves a high precision of 95.9%, its recall and AP rates of 80.5 and 85.6%, respectively, still fall short of the performance of MG-SNN combined with YOLOv5-x (98.3%). By capturing motion information, MG-SNN can more accurately identify and locate targets in dynamic scenes, effectively enhancing the contrast between targets and backgrounds. This enables the detection algorithm to more precisely separate and identify small targets. The CFINet and DyHead models, which are designed for small object detection, achieve AP values of 96.4 and 91.2%, respectively. However, the recall of CFINet is only 62.0%, lower than the performance of MG-SNN combined with YOLOv5-x (81.1%). Compared with other models, MG-SNN + YOLOv5-x achieves a competitive balance between computational complexity and real-time performance, with GFLOPs increasing slightly to 204.5 while maintaining a high FPS of 69.9, which demonstrates the integration’s ability to enhance detection capabilities without significant additional computational cost, making it suitable for dynamic, real-time applications. MG-SNN has proven to outperform other methods in detecting small-target drones within complex backgrounds. This is because it can extract motion information and integrate spatiotemporal features from historical data. Combining motion saliency extraction networks with advanced object detection networks significantly enhances overall object detection performance. This approach effectively utilizes spatiotemporal features from historical data to improve the detection of small drones in complex backgrounds.

**Table 5:** This is the result of a comparison experiment.

Methods	Precision (%)	Recall (%)	AP (%)	GFLOPs	FPS
YOLOv7-x	95.9	80.5	85.6	188.0	65.4
CFINet	93.4	62.0	71.4	111.57	38.6
DyHead	85.8	72.0	73.4	43.52	15.4
YOLOv5-x	95.4	77.4	82.8	203.8	64.5
MG-SNN+ YOLOv5-x	**98.3**	**81.1**	**86.1**	204.5	69.9

Bold values indicate the best performance for each metric within the respective method comparison.

### 4.4 Qualitative results of object detection

**Figure 9:**
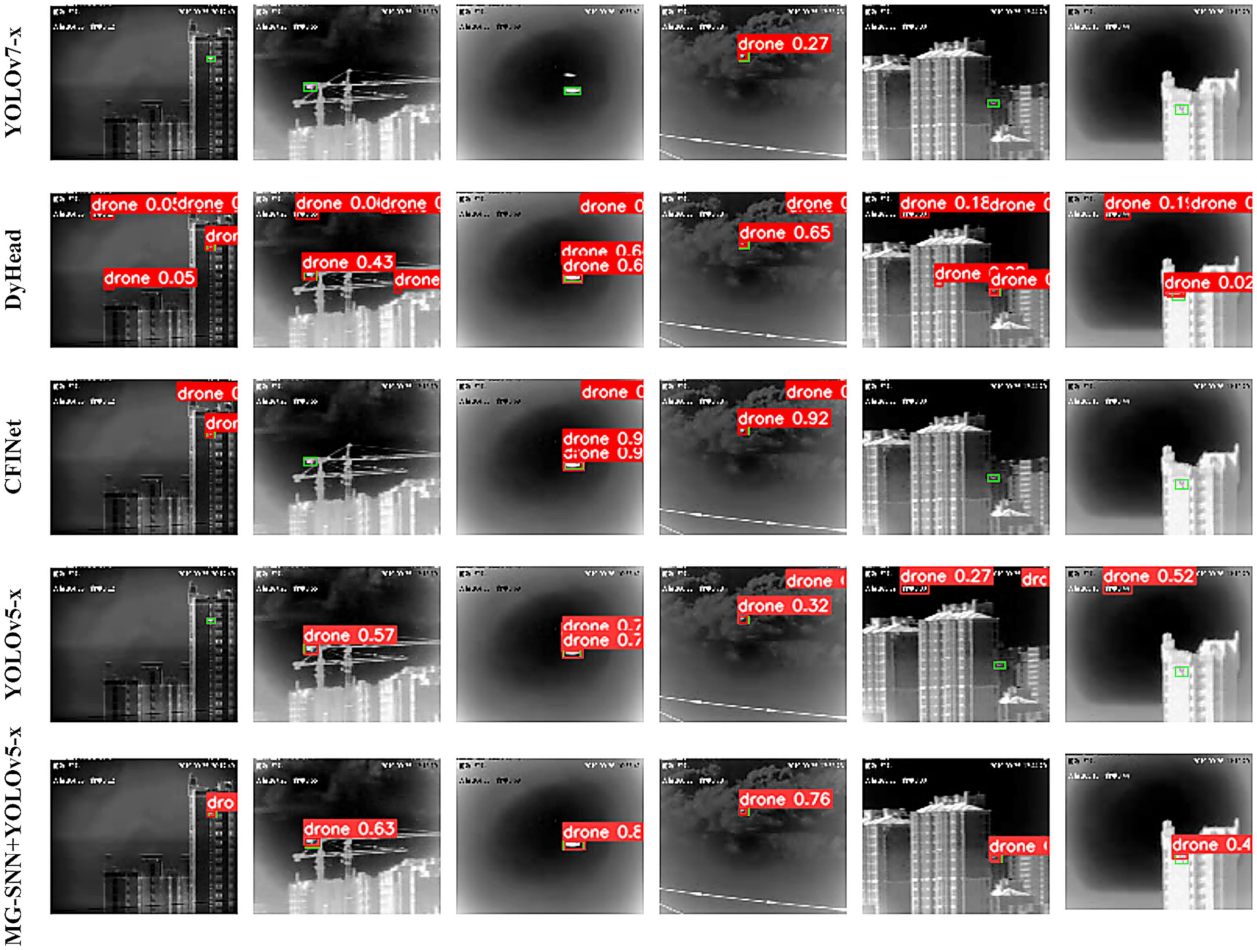
Visual comparison of test methods in test set videos of detection results. Green rectangles stand for the ground truth of object targets. Red rectangles represent detection results obtained by the test methods. The marked numbers are the confidence scores for the corresponding results.

The experimental results are shown in Figure 9. In the figure, green rectangles represent ground truth annotations, and red rectangles represent the detection result bounding boxes. The test data covers urban and cloud backgrounds, where drone targets are difficult to identify. In complex backgrounds, YOLOv5 can detect drone targets in most scenarios, but some bounding boxes do not fully overlap with the actual annotations, resulting in false detections. The CFINet and DyHead models fail to generate accurate detection results, missing the targets and producing erroneous detections. YOLOv7 fails to detect drone targets in several scenarios, indicating a tendency to miss small targets. By utilizing the motion saliency features extracted by the SNN and combining them with the response maps generated by YOLOv5, the target areas are significantly enhanced. Post-processing the spatiotemporal depth information of the video frames improves target recognition accuracy. This demonstrates that MG-SNN can be combined with other models for tasks such as object detection. By effectively integrating spatiotemporal information, the reliability and accuracy of detection are enhanced, providing stronger technical support for various practical applications.

## 5 Discussion

The MG-SNN has demonstrated outstanding performance in complex dynamic visual tasks, producing outputs that align with the processing of dynamic data in the primary visual cortex. It shows lower MAE in traditional performance evaluation metrics, validating its accuracy in extracting motion information. Compared to traditional convolutional neural networks and hybrid architectures, MG-SNN has demonstrated stable responses to dynamic targets with reduced iterations. The visual dynamic features extracted by MG-SNN have served as a motion guidance module, enhancing the object detection capabilities of small drones in complex backgrounds and enabling the deployment of drone feature extraction on neuromorphic hardware. Experimental results have indicated that the fused model outperforms the original model in terms of recognition accuracy and reliability. It can be flexibly integrated into existing object detection frameworks, effectively addressing the adaptability issues of traditional visual perception algorithms when handling fast-moving targets and complex backgrounds.

The two-layer MG-SNN model involves video frames passing through the photoreceptor input layer before the extracted features are transmitted to the ganglion cell output layer. During this process, spikes are fired when a neuron membrane voltage reaches a certain threshold, influenced by the input signals and synaptic weights, using neuron populations for information encoding helps mitigate noise. Even if individual neurons transmit erroneous information, the network as a whole can correct this deviation, reflecting the collective intelligence of biological neural systems.

The YOLO method is limited to processing single-frame information and neglects the handling of motion information in multi-frame inputs. Traditional artificial neural networks process only spatial information, while SNNs propagate spike times from presynaptic to postsynaptic neurons, thereby conveying temporal information. Other potential information in presynaptic neurons, which could provide valuable insights for the network, is discarded. Experimental results demonstrate that MG-SNN + YOLO achieves significant performance improvements over the baseline YOLO model, with an accuracy increase of 2.0–5.0%, a recall improvement of 0.2–3.7%, and an AP enhancement of 2.4–3.3%. Adding MG-SNN enhances the ability to capture deep spatiotemporal features, making the model more robust in distinguishing targets from complex backgrounds, leading to higher precision and recall, and stronger generalization capabilities. MG-SNN effectively compensates for the lack of motion information in handling complex dynamic scenes. Through advanced temporal processing, bio-inspired feature extraction, and spatiotemporal information computation, the combined architecture processes these scenes efficiently and enhances object detection accuracy. By integrating the MG-SNN motion guidance module with the YOLO framework, the system maintains real-time performance while improving detection capabilities, particularly for small targets in dynamic scenarios.

Future work will prioritize optimizing the current implementation of MG-SNN to enable seamless real-time integration for dynamic environments. Deploying MG-SNN on neuromorphic hardware optimized for event-driven and energy-efficient processing, such as Loihi or SpiNNaker, designed for event-driven and energy-efficient processing, will reduce computational overhead and latency, addressing current challenges in resource-constrained systems. Simplifying the MG-SNN architecture through model pruning and approximation will further enhance scalability, making the MG-SNN + YOLO framework more suitable for real-time detection tasks while maintaining accuracy and robustness in complex dynamic scenes, including improved resolution handling. In addition to improving real-time scalability, future research will explore the application of MG-SNN in swarms of small drones, transitioning from single-drone operations to collaborative multi-drone systems. This will involve integrating multi-source information, including pose estimation and data from lidar, RGB-D cameras, and inertial sensors, to enhance motion feature extraction and target detection in dynamic environments. Transitioning the framework towards online algorithms, incorporating event-based processing and real-time learning techniques will reduce memory consumption, computational overhead, and latency by optimizing the spiking neuron calculations within the current model. These improvements will also enhance system responsiveness and adaptability. With its advanced temporal processing, bio-inspired feature extraction, and combined spatio-temporal information computation, the MG-SNN framework has the potential to provide robust, scalable, and energy-efficient solutions for complex real-world scenarios, especially in resource-constrained systems and multi-drone platforms.

## 6 Conclusion

Achieving motion feature extraction and object detection for objects in terms of complex dynamic backgrounds and neuromorphic hardware deployment remains a challenging task. This study has delved into the potential of integrating the processing mechanisms of the biological retina with spiking neural networks (SNNs) for the first time. A two-layer pure SNN model, the Magno-Spiking Neural Network (MG-SNN), has been proposed to simulate the visual information transmission process and achieve motion feature outputs consistent with biological visual pathways as a motion feature extraction module for object detection tasks. A Visual-Magnocellular Dynamics Dataset (VMD) has been developed and a multi-frame spike temporal encoding strategy has been adopted to effectively extract and process dynamic visual information. By combining dynamic thresholds and the STDP rule, a Dynamic Threshold Multi-frame Spike Time Sequence (DT-MSTS) backpropagation method has been proposed to facilitate the extraction of motion features within the SNN architecture. Additionally, MG-SNN has been integrated with the YOLO model to design a retinal-inspired spiking neural network architecture for drone motion extraction and object detection. This study has demonstrated the synergistic advantages of retinal mechanisms and SNNs in visual information processing, highlighting the potential for advancing drone visual detection technology, explores the possibility of deploying neuromorphic chips in the form of software, and points towards future directions for managing complex spatiotemporal data in real-world object detection tasks. Future research will focus on expanding the applicability of MG-SNN to broader contexts, including collaborative multi-drone systems and dynamic, resource-constrained environments. Advancements such as the deployment of neuromorphic hardware, the development of efficient real-time algorithms, and the integration of multi-source information will further enhance the system scalability, robustness, and energy efficiency, and are expected to extend the MG-SNN to semantic segmentation or video tracking. These efforts aim to bridge the gap between experimental research and practical deployment, enabling applications in areas such as multi-drone coordination, large-scale surveillance, and disaster response.

## Data Availability

The datasets presented in this study can be found in online repositories. The names of the repository/repositories and accession number(s) can be found below: https://github.com/cecilia-zz-jy/MG-SNN.

## Footnotes

^1^ https://anti-uav.github.io/dataset/
