# Supplementary material for: Motion feature extraction using magnocellular-inspired spiking neural networks for drone detection
Source: Front Comput Neurosci. 2025 Jan 22;19:1452203. doi: 10.3389/fncom.2025.1452203 (PMC11794278; doi:10.3389/fncom.2025.1452203)
Supplement: Supplementary file 1 [file Table_1.DOCX]

Supplementary Material

# Spike Timing Dependent Plasticity

The connection strength between a pair of neurons can be determined by the relative timing of the spikes emitted by these two neurons. The relative timing of spikes between the presynaptic and postsynaptic neurons can be used to define the learning rule for updating synaptic weights$\Delta W$. $a$ denotes the maximum amount of synaptic modulation that occurs when the difference between the issuance times of the pre-neuron and post-neuron is close to zero, and $\tau$ is the spike update time constants:

$$\boldsymbol{\Delta W=}\boldsymbol{a}\boldsymbol{e}^{\boldsymbol{\Delta t/\tau}}$$

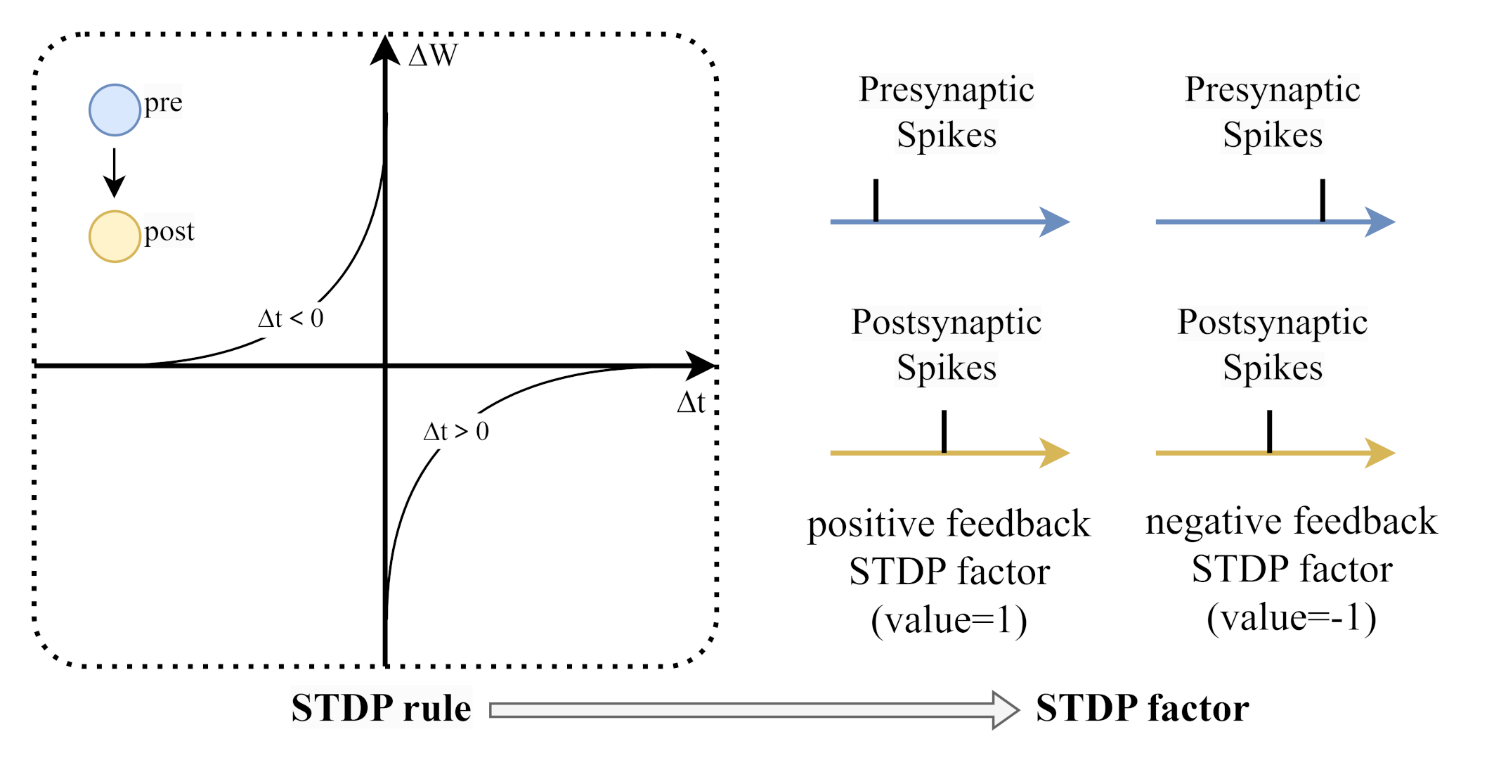


**Supplementary Figure 1.** The spike time difference, denoted as $\Delta t$, is the difference between the spike times of the pre-neuron and the post-neuron. The left side of the diagram shows the fitted STDP rule curve. Referring to the STDP rule of spiking neurons, we set the STDP factor to determine the weight updates, which aligns with the essence of the STDP rule. This factor controls the adjustment of synaptic weights based on the precise timing of spikes between connected neurons, reinforcing or weakening the synaptic connection accordingly.

# ANN neurons vs. SNN neurons

ANN neurons are an abstraction and simulation of the structure and function of biological neural systems. The output of an ANN neuron typically represents an analog value over a given interval, reflecting the continuous activation value received from the previous neuron. This value is transmitted through synapses to the dendrites and ultimately processed by the cell body. In ANNs, neurons use high-precision and continuous value-encoded activation values for communication with each other. However, spiking neurons, inspired by biological neural systems and also known as SNN neurons, respond to various changes in the internal and external environment by emitting sequences of spikes. This makes them more biologically interpretable. Communication between spiking neurons occurs through binary events rather than continuous activation values. As shown in Supplementary Figure 2(A), a typical single ANN neuron uses continuous activation values, while Supplementary Figure 2(B) depicts a single SNN neuron. Although its structure is similar to that of an ANN neuron, its behavior is different, relying on the timing and frequency of spikes to encode and transmit information.


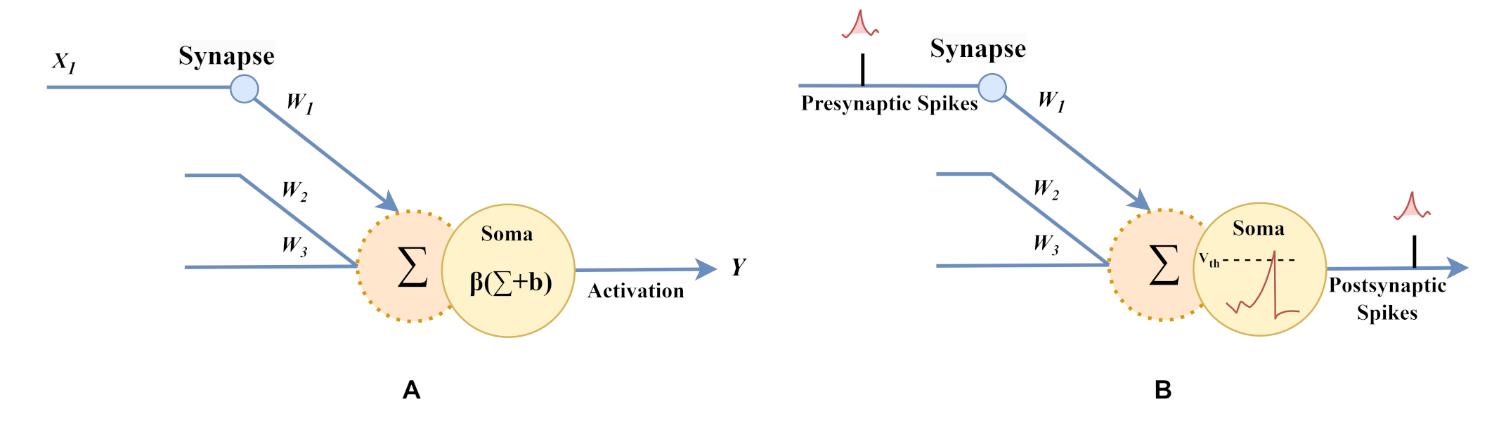


**Supplementary Figure 2.** (A) ANN neuron transmits spatial features with inputs of continuous activation; (B) SNN neuron contains spatio-temporal features characterizing information transfer in the form of discrete pulses with biological plausibility.

# Setting of IoU threshold

In our object detection experiments, we chose to use an IoU threshold of 0.25 and an initial image size of 128x128 to better accommodate small object detection tasks. For the MG-SNN model, due to the computational limitations previously mentioned, it can only accept an input and output size of 120x100. Therefore, during the feature fusion preprocessing stage, we redefined the MG-SNN size to 128x128 to meet the input requirements of the overall framework.

From the perspective of image size and salient box area, the majority of salient boxes in the Anti-UAV-2021 Challenge dataset and the Anti-UAV-2023 Challenge dataset occupy only about 0.3% of the image area, and the VMD dataset generated from them shows similar characteristics. Additionally, due to image size and software limitations of SNNs, the area occupied by small objects in the image is relatively small. Choosing a lower IoU threshold (such as 0.25) provides greater flexibility, allowing for a larger positional error between the detection box and the actual target, thereby reducing missed detections caused by slight misalignments of the detection box.

We have added the experimental results for an IoU threshold of 0.5, as shown in Supplementary Table 1. The results in the table demonstrate the improvement in target detection performance when using MG-SNN as a plug-and-play motion feature extraction module under different IoU thresholds. The IoU=0.25:0.95 represents the average precision (AP) calculated across multiple IoU thresholds (from 0.25 to 0.95 in 0.05 increments), providing a comprehensive assessment of model performance at varying levels of overlap.

For instance, the AP for MG-SNN+YOLOv6-l, MG-SNN+YOLOv5-s, and MG-SNN+YOLOv5-x increased to 85.0, 84.3, and 86.1, respectively, which are improvements of 2.6, 2.4, and 3.3 percentage points compared to the original models (IoU=0.25). Additionally, the MG-SNN module also showed significant performance improvements under more stringent IoU thresholds (such as IoU=0.5 and IoU=0.5:0.95). The AP for MG-SNN+YOLOv5-s and MG-SNN+YOLOv5-x were 40.3 and 40.1, respectively, representing improvements of 3.8 and 4.6 compared to using YOLOv5-s and YOLOv5-x alone (IoU=0.5:0.95).

**Supplementary Table 1.** Ablation study on the generalization of MG-SNN when applying to popular object detection methods at different IoU thresholds.

| **Methods** | **AP(IoU=0.25)** | **AP(IoU=0.25:0.95)** | **AP(IoU=0.5:0.95)** | **AP(IoU=0.5)** |
| --- | --- | --- | --- | --- |
| YOLOv6-l | 82.4 | 51.4 | 37.1 | 71.8 |
| MG-SNN+ YOLOv6-l | **85.0** | **52.7** | **38.6** | **72.5** |
| YOLOv5-s | 81.9 | 51.2 | 36.5 | 76.1 |
| MG-SNN+ YOLOv5-s | **84.3** | **54.1** | **40.3** | **80.3** |
| YOLOv5-x | 82.8 | 50.2 | 35.5 | 76.4 |
| MG-SNN+ YOLOv5-x | **86.1** | **54.3** | **40.1** | **80.5** |

In the comparison experiments, we selected IoU=0.25 as the primary evaluation metric to better reflect the model's performance in small object detection tasks. Small objects occupy fewer pixels in the image and are easily affected by background complexity, making a lower IoU threshold more suitable for evaluating the detection effectiveness of these targets. The results in the table show that the application of the MG-SNN module significantly improves detection performance across different models (IoU=0.25).

For example, the AP for MG-SNN + YOLOv5-x reaches 86.1 at IoU=0.25, an improvement of 3.3 percentage points over the YOLOv5-x model alone. While the MG-SNN module also improves performance at higher IoU thresholds, such as IoU=0.5, the increase is relatively smaller. At IoU=0.5, the AP for MG-SNN + YOLOv5-x is 80.5, only 4.1 percentage points higher than the original model. The more pronounced improvement in model performance at the IoU=0.25 threshold better reflects the MG-SNN module's advantages in enhancing the precision and recall of small object detection. Therefore, selecting IoU=0.25 as the primary evaluation metric not only allows for a better assessment of small object detection performance but also highlights the significant contribution of the MG-SNN module in this task.

# Comparison of F1-scores

F1-score is an important evaluation metric for the performance of classification models, representing the harmonic mean of precision and recall. Specifically, the F1-score takes into account both the precision (the accuracy of the model in predicting positive samples) and recall (the coverage of the model in identifying positive samples), providing a balanced evaluation criterion. The closer the F1-score is to 1, the better the model balances accuracy and coverage in predicting positive samples. The F1-score is calculated using the following formula:

| $F1=\frac{Precision\times Recall}{Precision+Recall}$ | (13) |
| --- | --- |

Supplementary Table 2 presents the F1-score results of different object detection methods after integrating the MG-SNN module. The advantage of the F1-score is that it balances the model's precision and recall, making it a more comprehensive evaluation metric, especially in cases of imbalanced class distribution. The results show that the F1-scores for MG-SNN+YOLOv6-l, MG-SNN+YOLOv5-s, and MG-SNN+YOLOv5-x increased to 87.5%, 86.7%, and 88.9%, representing improvements of 3.6, 0.9, and 3.4. This indicates that MG-SNN, as a motion feature extraction module, effectively enhances the model's precision and recall, thereby significantly improving the overall detection performance of the model in handling complex backgrounds and small object detection tasks.

**Supplementary Table 2.** F1-score on the generalization of MG-SNN when applying to popular object detection methods.

| **Methods** | **F1(%)** |
| --- | --- |
| YOLOv6-l | 83.9 |
| MG-SNN+ YOLOv6-l | **87.5** |
| YOLOv5-s | 85.8 |
| MG-SNN+ YOLOv5-s | **86.7** |
| YOLOv5-x | 85.5 |
| MG-SNN+ YOLOv5-x | **88.9** |

# Comparison of confusion matrix

The confusion matrix is used in object detection tasks to evaluate the detection and classification performance of a model, particularly for the detection accuracy and false positive rate of various targets. Specifically, the rows of the confusion matrix represent the actual labels, while the columns represent the predicted labels. By analyzing the confusion matrix, one can reveal the model True Positive Rate (TPR) and False Positive Rate (FPR), thereby assessing the model's detection capability and the incidence of false positives, and can highlight the degree of confusion between different categories, such as instances where a target of one category is misclassified as another category, and the issue of false negatives (FN), where actual targets are not detected.

The confusion matrix results (IoU=0.25) are shown in Supplementary Figure 3, where "Drone" represents the TPR, indicating the proportion of actual small drone targets correctly detected. "Background FN" represents missed targets. Integrating the MG-SNN module significantly enhanced the drone detection performance of YOLOv6-l, YOLOv5-s, and YOLOv5-x models. In the YOLOv6-l model, the introduction of MG-SNN reduced the background FN from 0.22 to 0.19. In the YOLOv5-x model, MG-SNN significantly improved the TPR for drone detection (from 0.80 to 0.82) and reduced the false positive rate (from 0.20 to 0.18), further proving the effectiveness of the MG-SNN module in enhancing detection accuracy and reducing false positives.

For the YOLOv5-s model, although the TPR for drone detection in the confusion matrix remains unchanged at 0.80, other performance metrics show significant improvements (Table 1, Table 2). The confusion matrix primarily focuses on whether detected targets are correctly classified, while Precision, Recall, and AP also consider the position and size of the detection boxes. The slight changes in TPR and FPR and the improvements in Precision and Recall compared to the original model indicate that the model has reduced false positives without significantly increasing false negatives.


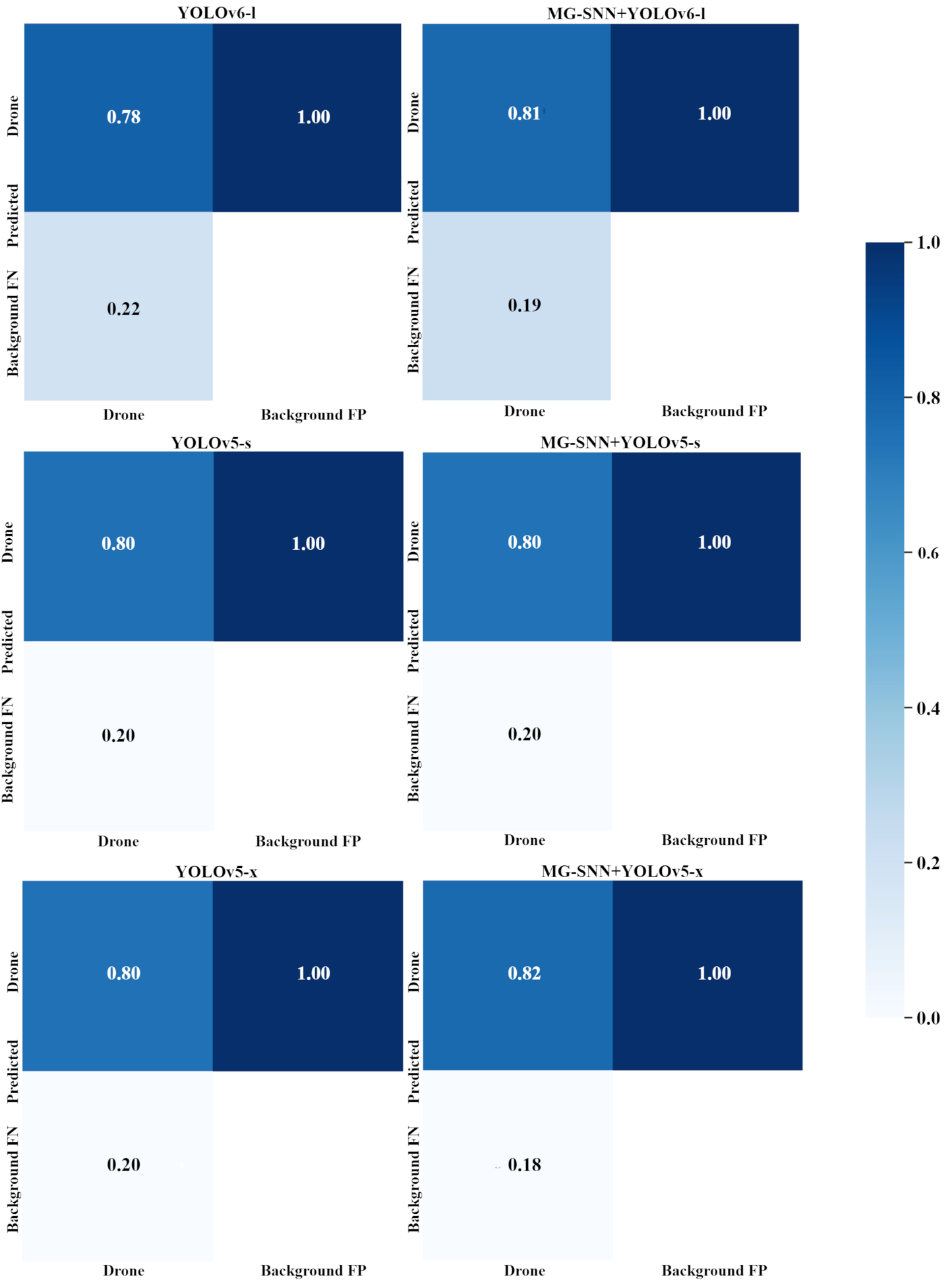


**Supplementary Figure 3.** The confusion matrix on the generalization of MG-SNN when applying to popular object detection methods.

# Feature Map Visualization

Supplementary Figure 4 illustrates the output results from the final layer of the MG-SNN + YOLOv5-x architecture. Each image contains 32 smaller images, representing heatmaps of the first 32 channels in the output layer. The circular or central focus of the activations suggests that the model is likely identifying features or objects that have a certain shape or motion pattern, corresponding to the characteristics of small drones.

The feature maps from the final layer of the MG-SNN + YOLOv5-x model demonstrate how the model focuses on different spatial regions and extracts diverse features critical for drone detection. The strong and varied activations indicate that the model is learning to recognize and localize small objects effectively, even in complex backgrounds. These visualizations highlight the effectiveness of the MG-SNN module in enhancing the model’s feature extraction process, particularly by focusing on motion-related and spatial features that contribute to improved detection performance. The observed sparsity and selectivity in activation patterns further suggest that the model is efficiently utilizing its capacity to focus on the most informative regions, supporting its capability in handling the task of small object detection.

The concentration of activations and the varied intensity patterns across the channels imply that the MG-SNN module helps the model extract motion-related and spatial features that are crucial for detecting small, fast-moving objects like drones. The relatively consistent yet varied activation patterns show that the module is aiding in distinguishing important motion cues from the background, thereby improving the model detection accuracy.


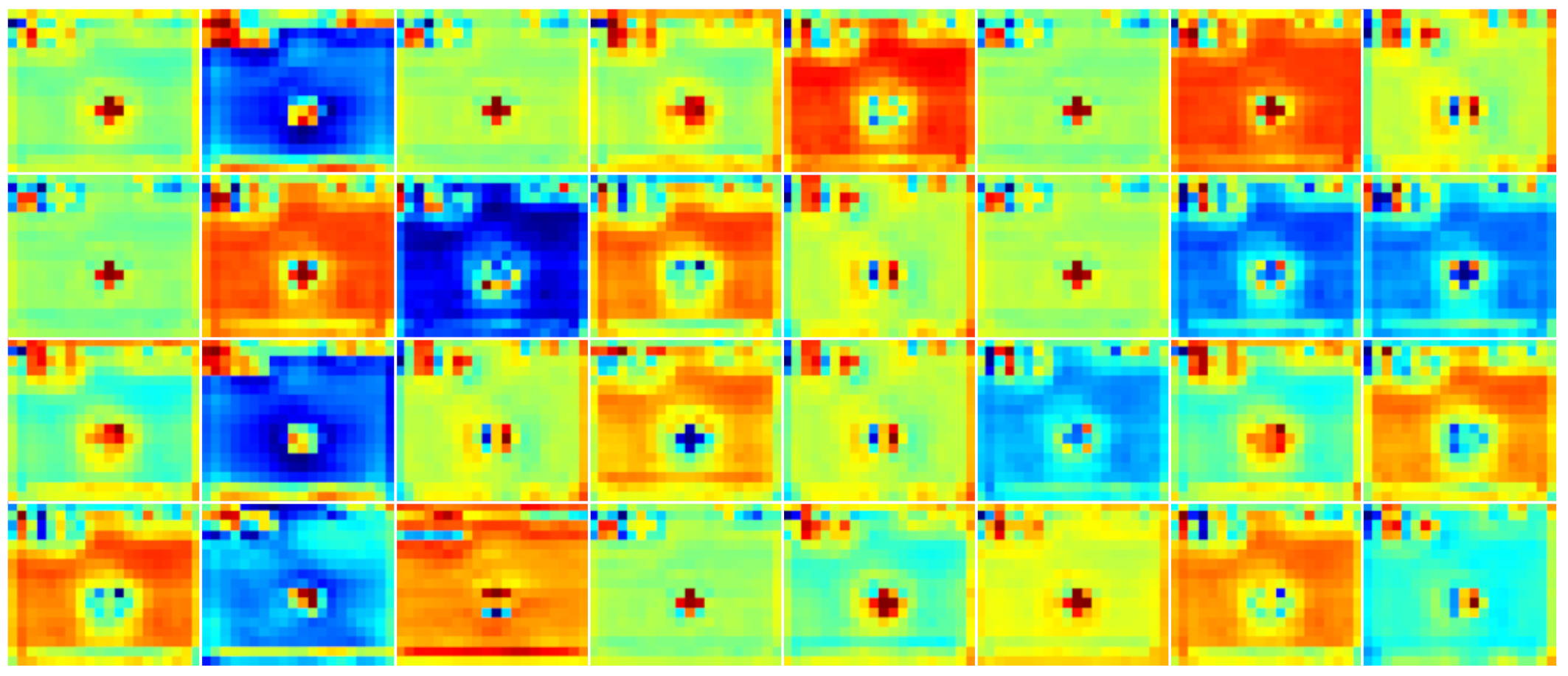


**Supplementary Figure 4.** The feature map on the generalization of MG-SNN when applying to popular object detection methods (Output layer).

# Discussion of the Superiority of Motion Feature Extraction in Terms of Biological Properties

The magnocellular pathway, involving amacrine cells, prioritizes recent inputs while diminishing historical information, which can lead to difficulty in effectively filtering sudden background changes in video or image processing. Given the model emphasis on recent information, newly appearing elements, such as background changes, are assigned greater importance. If these changes are transient or sudden, the model attenuation mechanism may fail to mitigate their impact, potentially causing significant effects on the final output. This challenge becomes particularly pronounced when the output of the magnocellular pathway computation model is used with tools such as the bioinspired module of OpenCV, which cannot eliminate background blur and jitter (Supplementary Figure 5A, Supplementary Figure 5B). MG-SNN avoids the limitations associated with amacrine cells and effectively focuses on key visual features. In applications such as drone flight, MG-SNN effectively mitigates background interference by accurately identifying key dynamic targets and combining the motion saliency feature sites extracted from MG-SNN with an advanced object detection network, which can significantly improve the overall object detection performance. In practical applications such as video surveillance and target tracking, implementing and combining MG-SNN facilitates accurate identification and highlighting of dynamic target objects while effectively suppressing irrelevant backgrounds. Considering its potential deployment on neuromorphic hardware in the future, the algorithm effectiveness and promise have been substantiated. In future work, we plan to continue optimizing the network architecture, particularly exploring the interaction and potential advantages of hybrid architectures and 3D convolution techniques in handling datasets with complex spatiotemporal relationships, aiming for higher performance and better generalization capability.


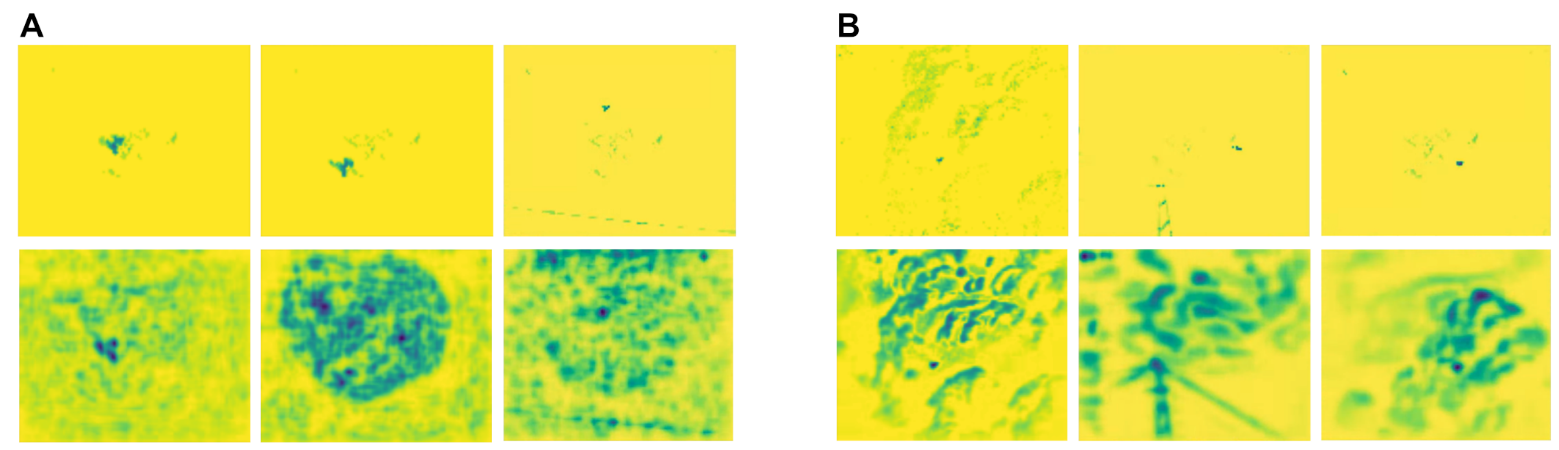


**Supplementary Figure 5.** (A) The issue of background blurring is produced by the output of the magnocellular pathway computational model. (B) The phenomenon of video frame skipping is produced by the output of the magnocellular pathway computational model; line 1 shows the output of MG-SNN; line 2 shows the output of the magnocellular pathway computational model.
